# Evaluating the ecological hypothesis: early life salivary microbiome assembly predicts dental caries in a longitudinal case-control study

**DOI:** 10.1186/s40168-022-01442-5

**Published:** 2022-12-26

**Authors:** Freida Blostein, Deesha Bhaumik, Elyse Davis, Elizabeth Salzman, Kerby Shedden, Melissa Duhaime, Kelly M. Bakulski, Daniel W. McNeil, Mary L. Marazita, Betsy Foxman

**Affiliations:** 1 Department of Epidemiology, University of Michigan School of Public Health, Ann Arbor, MI USA; 2 Department of Statistics, University of Michigan, Ann Arbor, MI USA; 3 Department of Biostatistics, University of Michigan School of Public Health, Ann Arbor, MI USA; 4 Department of Ecology and Evolutionary Biology, University of Michigan, Ann Arbor, MI USA; 5 Department of Psychology, West Virginia University, WVA, Morgantown, USA; 6 Department of Dental Practice & Rural Health, West Virginia University, Morgantown, WV USA; 7 Department of Oral and Craniofacial Sciences, Center for Craniofacial and Dental Genetics, School of Dental Medicine, University of Pittsburgh, Pittsburgh, PA USA; 8 Department of Human Genetics, Graduate School of Public Health, University of Pittsburgh, Pittsburgh, PA USA; 9 Clinical and Translational Sciences Institute, and Department of Psychiatry, School of Medicine, University of Pittsburgh, Pittsburgh, PA USA

**Keywords:** Oral microbiome, Early childhood, Ecological hypothesis, Early childhood caries, 16S rRNA gene, Whole genome shotgun metagenomics

## Abstract

**Background:**

Early childhood caries (ECC)—dental caries (cavities) occurring in primary teeth up to age 6 years—is a prevalent childhood oral disease with a microbial etiology. *Streptococcus mutans* was previously considered a primary cause, but recent research promotes the ecologic hypothesis, in which a dysbiosis in the oral microbial community leads to caries. In this incident, density sampled case-control study of 189 children followed from 2 months to 5 years, we use the salivary bacteriome to (1) prospectively test the ecological hypothesis of ECC in salivary bacteriome communities and (2) identify co-occurring salivary bacterial communities predicting future ECC.

**Results:**

Supervised classification of future ECC case status using salivary samples from age 12 months using bacteriome-wide data (AUC-ROC 0.78 95% CI (0.71–0.85)) predicts future ECC status before *S. mutans* can be detected. Dirichlet multinomial community state typing and co-occurrence network analysis identified similar robust and replicable groups of co-occurring taxa. Mean relative abundance of a *Haemophilus parainfluenzae/Neisseria/Fusobacterium periodonticum* group was lower in future ECC cases (0.14) than controls (0.23, *P* value < 0.001) in pre-incident visits, positively correlated with saliva pH (Pearson rho = 0.33, *P* value < 0.001) and reduced in individuals who had acquired *S. mutans* by the next study visit (0.13) versus those who did not (0.20, *P* value < 0.01). In a subset of whole genome shotgun sequenced samples (*n* = 30), case plaque had higher abundances of antibiotic production and resistance gene orthologs, including a major facilitator superfamily multidrug resistance transporter (MFS DHA2 family *P*_BH_ value = 1.9 × 10^−28^), lantibiotic transport system permease protein (*P*_BH_ value = 6.0 × 10^−6^) and bacitracin synthase I (*P*_BH_ value = 5.6 × 10^−6^). The oxidative phosphorylation KEGG pathway was enriched in case plaque (*P*_BH_ value = 1.2 × 10^−8^), while the ABC transporter pathway was depleted (*P*_BH_ value = 3.6 × 10^−3^).

**Conclusions:**

Early-life bacterial interactions predisposed children to ECC, supporting a time-dependent interpretation of the ecological hypothesis. Bacterial communities which assemble before 12 months of age can promote or inhibit an ecological succession to *S. mutans* dominance and cariogenesis. Intragenera competitions and intergenera cooperation between oral taxa may shape the emergence of these communities, providing points for preventive interventions.

Video Abstract

**Supplementary Information:**

The online version contains supplementary material available at 10.1186/s40168-022-01442-5.

## Background

In 2015–2016, 21% of US children aged 2–5 years showed evidence of early childhood caries (ECC), i.e., at least one primary tooth with one or more decayed, missing or filled tooth surfaces [1, 2]. ECC can be painful, may negatively impacts self-esteem, and is a strong predictor of future oral health problems [3, 4]. Microbial digestion of carbohydrates to acids which demineralize tooth enamel is the proximate cause [5–7]. Acid-producing bacteria, particularly *Streptococcus mutans* (*S. mutans*)*,* are frequently associated with ECC [5, 8]. No single bacterial species, however, has been conclusively identified as a necessary and sufficient cause of ECC across human populations [5, 8, 9]. Recent research emphasizes the ecologic hypothesis, which posits that overall shifts in the composition, structure, functional potential of the oral microbial community leads to dental decay [5, 10]. The oral microbiome assembles rapidly over the first 2 years of life [11]. However, few studies of ECC have prospectively tested the ecologic hypothesis during this early life period of assembly.

To assess the bacterial community in saliva and plaque samples, 16S rRNA gene amplicon sequencing is used to simultaneously measure many bacterial populations (although archaeal populations can also be measured using 16S amplicon sequencing, typical primers result in bias against archaea) [11–15]. However, common methods for analyzing 16S rRNA gene data fail to capture the spirit of the ecological hypothesis. Estimating the effect of each identified taxa as an independent predictor ignores how bacteria interact to affect risk, which is a key component of the ecological hypothesis [5, 16]. Diversity metrics, such as alpha and beta diversity, conveniently and efficiently summarize information across all measured taxa, but findings using associations between diversity metrics and cariogenesis are mixed [17–21]. The lack of consistency may be attributed to differences in study design, conduct and analysis, but also may reflect the inherent limitations of diversity metrics. These metrics ignore taxonomic, ecologic, and functional differences between bacteria which can impact disease processes such as cariogenesis [22]. Common methods for analyzing 16S rRNA gene data do not adequately encapsulate the ecologic hypothesis.

Microbial communities are dynamic, and early childhood is a susceptible life-period for short- and long-term oral microbial community assembly. The oral microbiome is acquired after birth and influenced by environmental factors [11, 12, 23]. Very few studies have prospectively tested the effect of oral microbial community assembly on ECC risk. A 2019 Australian study of 134 children followed for 5 years noted a shift in salivary microbiome composition at 39 and 48.6 months of age associated with future ECC [14]. Microbial taxa, including *Streptococcus sobrinus* and *Scardovia wiggsiae,* were identified as potential biomarkers of ECC onset. The percentage of *S. mutans* in saliva was the best prospective predictor of future ECC [13, 14]. The authors concluded, however, that the magnitude of change in the salivary microbiome was inadequate to differentiate between health and disease at clinical levels. A smaller 2020 study of 56 children aged 1–3 years followed for 2 years demonstrated that the early life salivary microbiome could prospectively classify future ECC onset (area under the receiver operating curve = 0.71) and identified several taxa that may serve as biomarkers of ECC [15]. These studies prospectively link community-wide shifts in the early-life salivary microbiome to ECC. However, they did not evaluate how co-occurrence or functional interactions between taxa influence ECC risk. Few longitudinal cohorts have explicitly evaluated how co-occurring groups of oral bacteria or functional interactions influence ECC risk.

To understand the influence of oral microbial community assembly on future oral health, explicit tests of the ecological hypothesis and identification of influential microbial populations is required. We used a longitudinal cohort of children to (1) prospectively test the ecological hypothesis of ECC in salivary bacterial communities and (2) identify co-occurring salivary bacterial populations influencing the risk of future ECC. We performed 16S rRNA gene amplicon sequencing on 855 longitudinal saliva samples from 99 children with ECC and 90 incidence-density sampled control children followed from 2 months to 5 years of age. We show that bacteriome-wide taxonomic information at 12 months of age better classifies future ECC status than *S. mutans* amplicon abundance alone. We identify robust and replicable communities of co-occurring bacteria using unsupervised clustering techniques, including a protective community of *Neisseria/Haemophilus parainfluenzae/Fusobacterium periodonticum* which was less abundant in future ECC cases. Finally, we comment on ecological and functional interactions that may shape the assembly of these communities using clinical data and functional potential measurements from a subcohort with shotgun metagenonomic sequencing data.

## Results

### Description of cohort

**Fig. 1:**
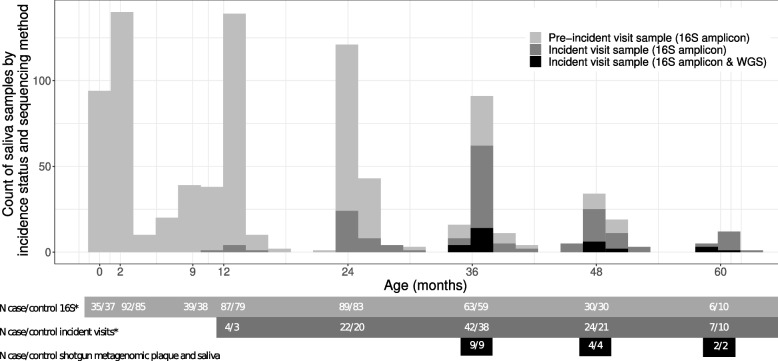
Histogram of available samples in this incidence-density case-control sample of 189 children from Appalachia. Children were followed from birth until 60 months of age, attending regularly scheduled study visits at birth (Pennsylvania children only), ~ 2 months, 1st-tooth emergence (Pennsylvania children only, average 9 months of age), ~ 12 months, ~ 24 months, ~ 36 months, ~ 48 months and ~ 60 months of age. Children who were diagnosed with white spots or enamel lesions were selected as cases. For each visit at which cases were diagnosed (incident-visit, dark-grey histogram), a similar number of controls were selected from the group of children free of white spots and enamel lesions at that time. Children could be selected as a control more than once or as a case and a control. In our sample, 1 child was selected as a control at both the 36- and 60-month visit, and 1 child was selected as a control at the 36-month visit and a case at the 60-month visit. The number of diagnosed cases/matched control children at each visit is shown in the dark-grey box and double counts these twice-sampled children at lines denoted with asterisk (*). All the available saliva samples from the incident-visit and all preceding visits for cases and selected controls were sequenced for the V4 region of the 16S rRNA gene (light-grey box and light-grey histogram). For a subsample of 15 children diagnosed with enamel lesions at or after the 36-month visit and their 15 matched controls both saliva and plaque samples from the visit of diagnosis/matching were shotgun metagenomic sequenced (black box and black histogram)

We selected an incidence density-matched case-control subset from the Center for Oral Health Research in Appalachia 2 (COHRA2) cohort. In the entire COHRA2 cohort, 47% of children were female, 79% were White and 71% were delivered vaginally. At 2 months of age, 58% of children were breastfed; this decreased to 32% by 12 months of age and 6% by 24 months of age. By 24 months of age, 3.8% of children in COHRA2 had a carious lesion or white spot. We analyzed a nested case-control sample of 99 children who developed a carious lesion or white spot at or before 60 months of age and 90 control children who were free of dental lesions at the age of case diagnosis (Additional file 1: Figure S1). Of the 189 children, 169 were White and 20 were bi- or multi-racial, 100 were from West Virginia and 89 from Pennsylvania, and 97 male and 92 female. None of these characteristics differed between cases and controls (Table 1). The mothers of controls were more likely to be educated beyond high school (63%) than the mothers of cases (33%, *P* < 0.001). Cases and controls were similar in the distribution of delivery mode, recent antibiotic exposure, breastfeeding, and count of erupted primary teeth (Table 1). Sampled controls were representative of the underlying disease-free cohort, although the proportion of bi- and multi-racial children was lower in the nested case-control sample (Additional file 2). Among the 99 ECC case children, the youngest age of diagnosis was 12 months, with a mean age of diagnosis of 38 months, additional information on case severity is presented in Additional files 3 and 4. We sequenced the V4 16S rRNA gene region in saliva samples from the visit corresponding to ECC diagnosis (incident visit) and all preceding visits (pre-incident visits) for case and control children (Fig. 1, Figure S1–3 in Additional files 1 and 5). From the 855 saliva samples across all incident and pre-incident visits, we identified 3194 amplicon sequence variants (ASVs). We labeled ASVs that did not classify to the species level with ASV numbers. Alpha diversity of the salivary microbiome increased as children aged. Alpha diversity was inconsistently associated with future ECC diagnosis across visits (Table 2). For a subcohort of 15 cases and matched controls, we also performed shotgun metagenomic sequencing on plaque and saliva samples from the visit corresponding to the time of case diagnosis (Additional files 1 and 6).

**Table 1:** Associations between future early childhood caries case status and sociodemographic and behavioral measures, among pre-incident children from Appalachia

	~2-month visit^**a**^			~12-month visit^**a**^			~24-month visit^**a**^		
	Case, *N* = 99^*1*^	Control, *N* = 91^*1*^	***p*** value^*2*^	Case, *N* = 89^*1*^	Control, *N* = 81^*1*^	***p*** value^*2*^	Case, *N* = 73^*1*^	Control, *N* = 69^*1*^	***p*** value^*2*^
Child's race			0.8			0.8			0.3
Bi- or multi-racial	10 (10%)	10 (11%)		8 (9.0%)	8 (9.9%)		5 (6.8%)	8 (12%)	
White	89 (90%)	81 (89%)		81 (91%)	73 (90%)		68 (93%)	61 (88%)	
Child's sex			> 0.9			> 0.9			0.7
Female	48 (48%)	44 (48%)		45 (51%)	41 (51%)		36 (49%)	32 (46%)	
Male	51 (52%)	47 (52%)		44 (49%)	40 (49%)		37 (51%)	37 (54%)	
Site			0.6			0.7			0.7
Pennsylvania	45 (45%)	45 (49%)		37 (42%)	36 (44%)		37 (50%)	37 (54%)	
West Virginia	54 (55%)	46 (51%)		52 (58%)	45 (56%)		37 (50%)	32 (46%)	
Currently breastfed			0.07			0.2			> 0.9
Currently breastfed	49 (49%)	57 (63%)		21 (24%)	27 (33%)		4 (5.5%)	4 (5.8%)	
Not currently breastfed	50 (51%)	34 (37%)		68 (76%)	54 (67%)		69 (95%)	65 (94%)	
Maternal report of child antibiotics within 3 months prior to visit	8 (8.1%)	8 (8.8%)	0.9	24 (27%)	18 (22%)	0.5	20 (27%)	23 (33%)	0.4
Count of primary teeth erupted	0.0 (0.3)	0.0 (0.0)	0.2	6.1 (3.0)	6.1 (2.8)	0.9	16.4 (1.9)	16.0 (1.8)	0.12
Delivery			0.5			0.7			0.4
C-section	35 (36%)	28 (31%)		33 (38%)	28 (35%)		28 (39%)	22 (32%)	
Vaginal	63 (64%)	63 (69%)		55 (62%)	53 (65%)		44 (61%)	47 (68%)	
Not reported	1	0		1	0		1	0	
Maternal education reported at prenatal visit			< 0.001			<0.001			< 0.001
Associates degree or higher	33 (33%)	57 (63%)		28 (31%)	51 (63%)		30 (41%)	48 (70%)	
High school degree or less	66 (67%)	34 (37%)		61 (69%)	30 (37%)		43 (59%)	21 (30%)	
In the past 7 days, about how often did your child drink fruit juice (juice composed of 100% fruit juice)?			0.8			0.003			0.04
Never or once	64 (94%)	47 (96%)		32 (37%)	53 (65%)		12 (17%)	21 (31%)	
Every few days	0 (0%)	0 (0%)		21 (24%)	10 (12%)		13 (19%)	20 (29%)	
Once a day	1 (1.5%)	1 (2.0%)		16 (18%)	10 (12%)		20 (29%)	11 (16%)	
Several times a day	3 (4.4%)	1 (2.0%)		18 (21%)	8 (10%)		24 (35%)	16 (24%)	
Missing or not reported	31	42		2	1		4	1	
Child’s teeth brushed/wiped?									
No	1 (100%)	1 (100%)		8 (9.4%)	12 (15%)	0.3	1 (1.4%)	1 (1.4%)	> 0.9
Yes, with toothpaste				35 (41%)	24 (30%)		65 (94%)	65 (94%)	
Yes, without toothpaste				42 (49%)	43 (54%)		3 (4.3%)	3 (4.3%)	
Missing or not reported	98	90		4	2		4	0	

^*1*^ Mean (SD); *n* (%)

^*2*^ Wilcoxon rank sum test; Fisher's exact test; Pearson’s chi-squared test

^*a*^ Includes duplicate records for 1 child selected as a control at 36 months and a case at 60 months, and 1 child selected as a control for both 36 and 60 month risk sets. Excludes samples from children diagnosed as a case at that visit and their corresponding risk-set controls (*N* = 6 at 12 months, *N* = 37 at 24 months)

**Table 2:** Associations between future early childhood caries and salivary microbiome measures, among pre-incident children from Appalachia

Characteristic	~2-month visit^**a**^			~12-month visit^**a**^			~24-month visit^**a**^		
	Case, *N* = 99^*1*^	Control, *N* = 91^*1*^	***p*** value^*2*^	Case, *N* = 89^*1*^	Control, *N* = 81^*1*^	***p*** value^*2*^	Case, *N* = 73^*1*^	Control, *N* = 69^*1*^	***p*** value^*2*^
Shannon	2.1 (0.5)	1.9 (0.5)	0.01	2.9 (0.4)	3.0 (0.4)	0.05	3.5 (0.3)	3.5 (0.4)	0.4
Missing	7	6		5	5		4	4	
Chao1	31.8 (12.3)	27.8 (10.0)	0.03	57.4 (16.8)	62.7 (14.6)	0.05	85.9 (19.3))	88.1 (19.2)	0.5
Missing	7	6		5	5		4	4	
*S. mutans* abundance	0.0 (0.0)	0.0 (0.0)	> 0.9	0.0 (0.0)	0.0 (0.0)	0.07	0.0 (0.0)	0.0 (0.0)	< 0.001
Missing	7	6		5	5		4	4	
*S. mutans* ASV detected			> 0.9			0.12			< 0.001
No	90 (98%)	83 (98%)		78 (93%)	75 (99%)		50 (72%)	64 (98%)	
Yes	2 (2.2%)	2 (2.4%)		6 (7.1%)	1 (1.3%)		19 (28%)	1 (1.5%)	
Missing	7	6		5	5		4	4	
*S. wiggsiae* abundance	0.0 (0.0)	0.0 (0.0)	0.7	0.0 (0.0)	0.0 (0.0)		0.0 (0.0)	0.0 (0.0)	0.3
Missing	7	6		5	5		4	4	
*S. wiggsiae* ASV detected			0.7						> 0.9
No	86 (93%)	78 (92%)		84 (100%)	76 (100%)		68 (99%)	65 (100%)	
Yes	6 (6.5%)	7 (8.2%)					1 (1.4%)	0 (0%)	
Missing	7	6		5	5		4	4	

^*1*^ Mean (SD); *n* (%)

^*2*^ Wilcoxon rank sum test; Fisher’s exact test; Pearson’s chi-squared test

^*a*^ Includes duplicate records for 1 child selected as a control at 36 months and a case at 60 months, and 1 child selected as a control for both 36- and 60-month risk sets. Excludes samples from children diagnosed as a case at that visit and their corresponding risk-set controls (*N* = 6 at 12 months, *N* = 37 at 24 months)

### *S. mutans* did not associate with future ECC diagnosis before 24 months of age, but was elevated in cases at the visit of first ECC diagnosis

A single ASV identified as *S. mutans.* We validated the identity of this ASV using BLAST and shotgun metagenomic sequencing data (Additional file 7; Additional file 1: Figure S4). At the 2- and 12-month visits, *S. mutans* was rare and not associated with future ECC diagnosis (Table 2). By the 24-month visit, *S. mutans* was more prevalent in future cases (Table 2; *P* value < 0.001). *S. mutans* prevalence and abundance was elevated in cases at the visit of ECC diagnosis: 13 of 20 ECC cases diagnosed at 24 months had *S. mutans* at the 24-month visit vs 2 of 18 matched controls (Additional file 8; *P* value = 0.001). Similarly, *Scardovia wiggsiae* was elevated at the visit of ECC diagnosis but not in visits preceding diagnosis (Table 2, Additional file 8).

### At 12 and 24 months of age, supervised random forest using the salivary bacteriome can predict ECC status before *S. mutans* detection

We investigated whether future ECC status could be predicted from a random forest classifier using the 273 most abundant and prevalent ASVs sequenced from saliva samples. Separate classifiers were built using samples from the 12- and 24-month visits. Only pre-incident samples were used, i.e., we predicted if a child would go on to be diagnosed with white spots or cavities at any of the 24-, 36-, 48-, or 60-month visits using their 12-month saliva sample. Children who were diagnosed with white spots or cavities at the 12-month visit and their incidence density-matched controls were excluded from the classifier. Similarly, we predicted if a child would go on to be diagnosed at any of the 36-, 48-, or 60-month visits using their 24-month saliva sample, excluding saliva samples from children diagnosed at 12 or 24 months. Thus, each random forest classifier predicted future ECC diagnosis using saliva samples from before disease was clinically apparent and diagnosed.

**Fig. 2:**
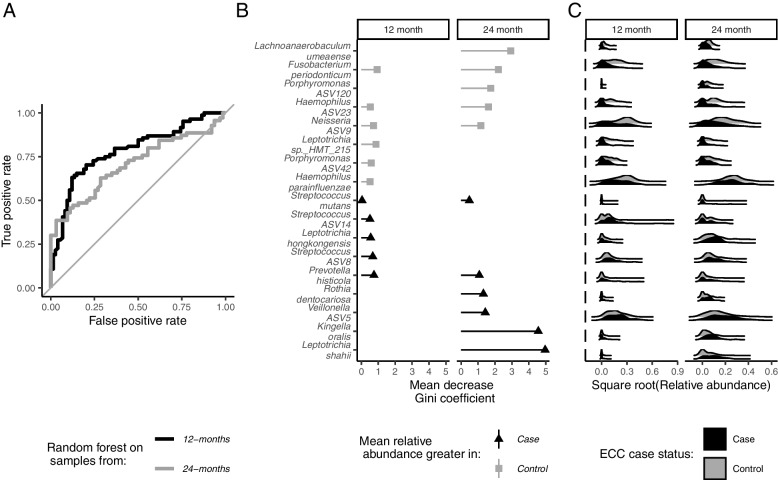
Taxa-wide supervised 5-repeat, 10-fold random forest classification models predict future early childhood caries status when using 12- (*n* = 158) and 24-month (*n* = 133) 16S rRNA gene amplicon sequenced saliva samples of children from Appalachia in an incidence density sampled case-control study (Center for Oral Health Research in Appalachia 2 cohort). **A** Area under the curve receiver operating curves from supervised random forests predicting future early childhood caries using the 273 most prevalent and abundant amplicon sequence variants at 12 months (black line) and 24 months (grey line). **B** Importance plots showing the top ten most important amplicon sequence variants from the 12- and 24-month supervised random forest classifiers performed on 273 amplicon sequence variants, as determined by mean decrease in the Gini coefficient, with the importance of the *S. mutans* amplicon included for comparison. **C** Joy plots showing the relative abundance distribution of the top 10 most important amplicon sequence variants and *S. mutans* among cases (black) and controls (grey) at the 12- and 24-month visits

The random forest using 273 ASVs showed good classification of future ECC status at the 12-month (AUC (95% CI): 0.78, (0.71–0.85)) and 24-month visits (AUC (95% CI) 0.72, (0.63–0.81)) (Fig. 2A). The mean decrease in the Gini coefficient provides a measure of how important a feature is for classification, with a larger decrease corresponding to a greater importance. In Fig. 2B, we show the 10 ASVs with the largest decrease in Gini coefficient from the 12- and 24-month supervised random forest classifiers. The Gini coefficient for *Streptococcus mutans* is included for comparison. In Fig. 2C, the distribution of the square root of ASV abundance is shown for cases (black) and controls (grey) for the ASVs with the largest decreases in Gini coefficient at the 12- and 24-month visits. Several of the important features from the random forest classifiers were more abundant in controls than in ECC cases (protective ASVs). Protective ASVs *Fusobacterium periodonticum* and *Neisseria* ASV9 were among the top 10 most important features in both the 12- and 24-month classifiers. *Haemophilus parainfluenzae* and *Porphyromonas* ASV42 were among the top 10 most important features in only the 12-month classifier while *Lachnoanaerobaculum umeaense* and *Porphyromonas ASV120* were among the top 10 most important features in only the 24-month classifier. Other important features were more abundant in ECC cases (cariogenic ASVs). Of these, only *Prevotella histicola* was among the top 10 most important features in both the 12- and 24-month classifier. Two *Streptococcus* ASVs were among the top 10 most important features in the 12-month classifier, but neither were identified as *S. mutans. Streptococcus* ASV8 was likely *Streptococcus salivarius*. Streptococcus ASV14 was closely related to *Streptococcus lactarius/peroris* (Additional file 7; Additional file 1: Figure S4).

### Unsupervised clustering techniques identify similar groups of co-occurring taxa, which associate with ECC

Next, we attempted to identify ecologically meaningful groups of co-occurring taxa. To do so, we used two different unsupervised clustering techniques. One technique, Dirichlet multinomial community state typing, groups together samples with similar distributions of taxa into discrete clusters or community state types (CSTs). Thus, each sample is assigned to a single CST. The other technique, weighted co-occurrence network analysis, groups together taxa which co-occur across samples using graphs. ASVs are network nodes joined by edges weighted by the frequency and correlation strength at which two nodes co-occur across samples. Clusters of co-occurring ASVs, or network modules, are identified from the graph.

**Fig. 3:**
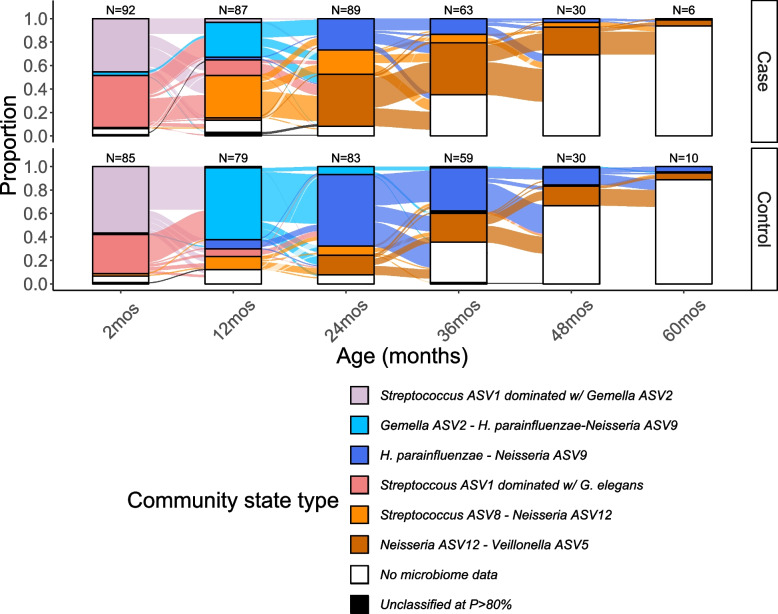
Community state typing clusters samples into 6 community state types (colors) corresponding to age (*x*-axis) and early childhood caries case status (facets) when performed on 855 longitudinal, 16S rRNA gene sequenced pre-incident and incident saliva samples from 189 children from Appalachia (191 records) in an incidence-sampled case-control study (Center for Oral Health Research in Appalachia 2 study). Alluvial plot showing the proportion of the sample in each community state type at each visit and the transitions between visits, faceted by early childhood caries case status. Bars are annotated at the top with the sample size N of cases and controls (excluding children who missed visit or did not have cleaned 16S rRNA gene amplicon saliva data for that visit). Only samples from the visit at which a case was diagnosed and preceding samples were sequenced, thus fewer samples were available at later ages

Using Dirichlet multinomial community state typing, we identified 6 community state types (CSTs) (Fig. 3, Additional file 1: Figure S5–6). We named CSTs after the ASVs defining their separation. CSTs corresponded to child age and ECC status. At the 2-month visit, most children’s samples belonged to one of two *Streptococcus*-dominated CSTs. Similar proportions of case and control samples were assigned to these two CSTs. At the 12-month visit, most control samples belonged to a more diverse *H. parainfluenzae-Neisseria ASV9–Gemella ASV2* CST while most cases samples belonged to a Streptococcus *ASV8–Neisseria ASV12* CST (Fig. 3). By the 24-month visit, most control samples transitioned to a second *Hemophilus parainfluenzae* and *Neisseria* ASV9 CST, while most case samples transitioned to a *Neisseria ASV12–Veillonella ASV5* CST (Fig. 3, Additional files 8 and 9). The odds of future ECC diagnosis were 8 (95%CI: (3, 22)) times higher for children assigned to the *Streptococcus ASV8-Neisseria ASV12* CST as compared to children assigned to the *H. parainfluenzae-Neisseria ASV9-Gemella ASV2* CST at 12 months after controlling for maternal education, count of emerged primary teeth, mode of birth delivery, breastfeeding, antibiotic exposure within 3 months and visit of case diagnosis (*P* value < 0.001, Table 3). Similarly, the odds future ECC diagnosis were 5 (95% CI (2, 12)) times higher for children assigned to the *Neisseria ASV12-Veillonella ASV5* CST as compared to those assigned to the *H. parainfluenzae-Neisseria ASV9* at 24 months, after controlling for maternal education, count of emerged primary teeth, mode of birth delivery, breastfeeding, antibiotic exposure within 3 months and visit of case diagnosis (*P* value < 0.001, Table 3).

**Table 3:** Odds ratios (ORs) and 95% confidence intervals (CIs) for community state types (CSTs) and future early childhood caries case status from logistic regressions stratified by visit (12- or 24-month visit) among 189 children in a nested case-control study selected from the Center for Oral Health Research in Appalachia 2 cohort study

Characteristic	Unadjusted					Adjusted^**1**^					Sensitivity^**2**^				
	***N***^**3**^	Case ***N***	OR	95% CI	***p*** value	***N***^**3**^	Case ***N***	OR	95% CI	***p*** value	***N***^**4**^	Case ***N***	OR	95% CI	***p*** value
**12-month visit CST**					< 0.001					< 0.001					< 0.001
*Gemella ASV2-H. parainfluenzae-Neisseria ASV9*	81	28	—	—		81	28	—	—		80	27	—	—	
*H. parainfluenzae-Neisseria ASV9*	9	2	0.54	0.08, 2.42		9	2	0.39	0.05, 2.03		8	2	0.28	0.03, 1.82	
*Streptoccous ASV1 dominated w/ G. elegans*	18	12	3.79	1.32, 11.9		18	12	4.32	1.34, 15.4		17	11	4.93	1.40, 19.1	
*Streptococcus ASV8-Neisseria ASV12*	42	33	6.94	3.02, 17.3		42	33	7.67	2.97, 21.8		40	31	8.18	3.00, 24.7	
*Neisseria ASV12-Veillonella ASV5*	2	2	NR^5^	NR^5^		2	2	--	--		2	2	NR^5^	NR^5^	
*Streptococcus ASV1 dominated w/ Gemella ASV2*	4	3	NR^5^	NR^5^		4	3	--	--		4	3	NR^5^	NR^5^	
**24-month visit CST**					< 0.001					< 0.001					< 0.001
*H. parainfluenzae-Neisseria ASV9*	67	24	Ref	—		67	24	—	—		66	23	—	—	
*Streptococcus ASV8-Neisseria ASV12*	20	14	4.18	1.48, 13.1		20	14	3.34	1.09, 11.2		20	14	4.93	1.44, 18.8	
*Neisseria ASV12-Veillonella ASV5*	40	30	5.38	2.31, 13.4		40	30	4.71	1.91, 12.4		37	28	7.12	2.57, 22.0	
*Gemella ASV2-H. parainfluenzae-Neisseria ASV9*	6	0	NR^5^	NR^5^		6	0	0.00			6	0	NR^5^	NR^5^	

^*1*^ Model adjusted for maternal education, count of emerged primary teeth, breastfeeding, antibiotic exposure within 3 months and visit of case diagnosis

^2^Model adjusted for all variables listed above + weekly frequency child consumes fruit juice (beverage composed of 100% fruit juice) and brushing/wiping of child’s teeth

^3^To ensure the salivary bacteriome is prospectively predicting future early childhood caries diagnosis, cases which were diagnosed at 12 months and corresponding controls (*N* = 6) were excluded from 12-month models. Similarly, cases and diagnosed at 24 months and corresponding controls (*N* = 37 at 24 months) were excluded from 24-month models. Includes duplicate records for 1 child selected as a control at 36 months and a case at 60 months, and 1 child selected as a control for both 36- and 60-month risk sets. 1 child with missing birth delivery mode excluded. *N* = 3 children with unassigned CST at 12 months

^4^Sample size as described in footnote 3, but additionally children with missing dietary or oral hygiene data (*n* = 5 at 12 months, *n* = 4 at 24 months) were excluded

^5^Because community state type assignment correlated with sample age, some community state types had very small cell counts at the 12- and 24-month visits. If a cell count for a community state type was < 10, we do not report the ORs or 95% CI  (not reported, NR) since these estimates are likely unstable. We use the CST with the largest cell count as the reference category in each visit strata

**Fig. 4:**
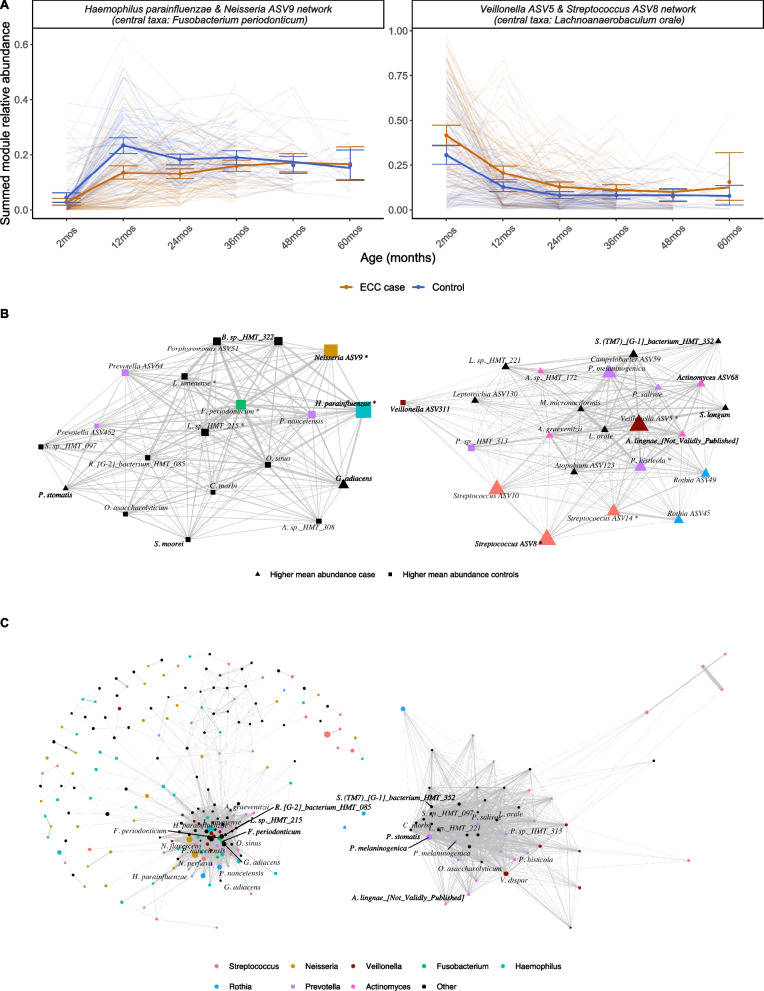
Weighted co-occurrence network graphs identifies two clusters of co-occurring taxa which were associated with age and early childhood caries case status among 855 longitudinal, 16S rRNA gene sequenced saliva samples from 189 children from Appalachia (191 records) in an incidence-sampled case-control study (Center for Oral Health Research in Appalachia 2 (COHRA2) study) and were reproducible in an independent longitudinal cohort of similarly aged children with a 10% prevalence of early childhood caries. **A** Spaghetti plots showing the summed module relative abundance of two of the five identified network modules from weighted co-occurrence networks. Networks were named using the two most abundant amplicon sequence variants in the network and the most central amplicon sequence variant. Summed module relative abundance calculated by summing the relative abundance of all amplicon sequence variants assigned to the same cluster. Thin, transparent lines are individuals over time, thick lines represent smoothed means, dots and bars are mean and bootstrapped 95% confidence intervals at each visit, including both pre-incident and incident visits. **B** Network graphs of the two network modules shown in **A**. On the left, the protective network module was dominated by *H. parainfluenzae* (turquoise) and a *Neisseria* taxon (gold), with central taxon *Fusobacterium periodonticum* (green). On the right, the cariogenic network module was dominated by *Streptococcus* (red) and *Veillonella* (brown), with additional *Actinomyces* (pink) and *Prevotella* (purple) members. Amplicon sequence variants (nodes) that were more abundant in cases are shown as triangles, those more abundant in controls are shown as squares. Larger nodes represent more abundant amplicon sequence variants, and nodes are colored by genus. Amplicon sequence variants which were among the top 10 most important features in the supervised random forests are annotated with an asterisk, *. Thicker edges represent stronger correlations between amplicon sequence variants. **C** Repeating the co-occurrence graph analysis in an independent longitudinal cohort of similarly aged children (Holgerson et al.) identified a *Haemophilus* (turquoise) and *Neisseria* (gold) dominated network with central taxon *Fusobacterium periodonticum,* similar in composition and structure to the protective network module identified in COHRA2. A *Veillonella dispar* (brown), *Prevotella* (purple), and *Streptococcus* (red) network module was similar in composition to the cariogenic network module identified in COHRA2. Larger nodes represent more abundant amplicon sequence variants, and nodes are colored by genus. Amplicon sequence variants which were shared between the corresponding modules in COHRA2 and the Holgerson et al. cohort are annotated. For visualization purposes, edges with weights < 0.03 were not included

Using weighted co-occurrence network analysis, we identified five network modules of co-occurring ASVs. Network modules were named after the top 2 most abundant ASVs in the network and the most highly connected or central ASV in the module (Fig. 4A, B; Figures S7–8 Additional files 1 and 10). We create a single summary measure for each network module by summing the relative abundance of all taxa assigned to the module. A *Haemophilus parainfluenzae* and *Neisseria* ASV9 network module with a *Fusobacterium periodonticum* as the most central taxa was more abundant in controls at 12 and 24 months. For every 1 percentage point increase in relative abundance of this network module at 12 months, the odds of ECC at a future visit were 0.94 (95% CI 0.91, 0.97) times higher, after controlling for maternal education, count of emerged primary teeth, breastfeeding, antibiotic exposure within 3 months, and visit of case diagnosis (*P* value < 0.0001, Table 4). Conversely, a *Veillonella* ASV5 and *Streptococcus* ASV8 network module with a central taxon of *Lachnoaerobaculum orale* was more abundant in cases (Fig. 4B). For every 1 percentage point increase in relative abundance of this network module at 12 months, the odds of ECC at a future visit were 1.04 (95% CI (1.02, 1.07)) times higher, after controlling for maternal education, count of emerged primary teeth, breastfeeding, antibiotic exposure within 3 months and visit of case diagnosis (*P* value = 0.001, Table 4). Three other network modules were not consistently associated with dental decay (Figure S7–8, Additional file 1). *S. mutans* was a member of one of these networks, which had *Streptococcus* ASV1 and *Neisseria* ASV12 as the most abundant ASVS and *Actinomyces* ASV41 as the most central.

**Table 4:** Odds ratios (OR) and 95% confidence intervals (CIs) for summed network module abundance and future early childhood caries case status from logistic regressions stratified by visit (12- or 24-month visit) among 189 children in a nested case-control study selected from the Center for Oral Health Research in Appalachia 2 cohort study

Characteristic	Unadjusted					Adjusted^**1**^					Sensitivity^**2**^				
	***N***^**3**^	Case ***N***	OR^**4**^	95% CI^*4*^	***p*** value	***N***^**3**^	Case ***N***	OR^*4*^	95% CI^*4*^	***p*** value	***N***^**5**^	Case N	OR^*4*^	95% CI^*4*^	***p*** value
**12-month saliva samples**															
*Veillonella ASV5 and Streptococcus ASV8 network*	159	83	1.04	1.01, 1.06	0.002	159	83	1.04	1.02, 1.07	< 0.001	154	79	1.04	1.02, 1.07	< 0.001
*Haemophilus parainfluenzae and Neisseria ASV9 network*	159	83	0.94	0.92, 0.97	< 0.001	159	83	0.94	0.91, 0.97	< 0.001	154	79	0.94	0.91, 0.97	< 0.001
**24-month saliva samples**															
*Veillonella ASV5 and Streptococcus ASV8 network*	133	68	1.04	1.01, 1.08	0.008	133	68	1.05	1.01, 1.09	0.009	129	65	1.04	1.01, 1.09	0.019
*Haemophilus parainfluenzae and Neisseria ASV9 network*	133	68	0.95	0.92, 0.99	0.016	133	68	0.96	0.92, 1.00	0.043	129	65	0.95	0.91, 1.00	0.033

^*1*^ Model adjusted for: maternal education, count of emerged primary teeth, breastfeeding, antibiotic exposure within 3 months and visit of case diagnosis

^2:^ Model adjusted for: all variables listed above + weekly frequency child consumes fruit juice (beverage composed of 100% fruit juice) and brushing/wiping of child’s teeth

^3^To ensure the salivary bacteriome is prospectively predicting future early childhood caries diagnosis, cases which were diagnosed at 12 months and corresponding controls (*N* = 6) were excluded from 12-month models. Similarly, cases and diagnosed at 24 months and corresponding controls (*N* = 37 at 24 months) were excluded from 24-month models. Includes duplicate records for 1 child selected as a control at 36 months and a case at 60 months, and 1 child selected as a control for both 36- and 60-month risk sets. 1 child with missing delivery excluded

^4^*OR* odds ratio, *CI* confidence interval

^5^ Sample size as described in footnote 3, but additionally children with missing dietary or oral hygiene data (*n* = 5 at 12 months, *n* = 4 at 24 months) were excluded

Although one unsupervised method clustered samples and the other clustered taxa, they identified similar clinically relevant patterns in bacterial compositional data. Both identified a pattern of *H. parainfluenzae* and *Neisseria* co-occurrence elevated in controls, and a pattern of *Streptococcus* and *Veillonella* elevated in cases. Nine of the ten ASVs used to name the networks (top 2 most abundant ASVs in each of five network modules) were also in the top 10 most important ASVs for defining the separation of CSTs (Additional file 1: Figure S9).

The ECC-associated communities identified through unsupervised clustering were robust to varying hyperparameters. In the CST analysis, we varied the number of k CSTs (*k* = 4 vs 5 vs 6, Additional file 11, Additional file 1: Figure S10). In the network analysis, we varied the normalization transform function (Hellinger vs center-log, Additional file 1: Figure S11). We also performed a sensitivity analysis to determine if these associations were robust to adjustment for cariogenic food consumption and oral hygiene. Very few children were eating or drinking high-sugar foods or beverages at 12 months of age, except for fruit juice (Additional file 12). The association between the CSTs and future ECC diagnosis remained unchanged when controlling for fruit juice consumption and tooth brushing/wiping in a sensitivity analysis (Table 3), as did the association between the network modules and future ECC diagnosis (Table 4).

### Communities identified through unsupervised clustering are reproducible in an external cohort

To examine the reproducibility of these bacterial community networks, we performed the same analytic pipeline (see “Methods” section) on publicly available 16S rRNA gene sequencing data from longitudinal saliva samples of similarly aged children with a 10% prevalence of early childhood caries (Holgerson et al.; PRJEB35824 [12];). We were unable to obtain access to metadata for these samples.

A *Haemophilus parainfluenzae* and *Neisseria perflava* network module with central taxa *Fusobacterium periodonticum* was also identified in the Holgerson et al. sample (Fig. 4C Additional file 1: Figures S12–13). The *Neisseria* ASV9 amplicon from our cohort was closely related to the *Neisseria perflava* amplicon from the Holgerson et al. cohort (Additional file 1: Figure S14A).

A similar *Veillonella dispar/Streptococcus/Prevotella* network module was also identified in the Holgerson et al. sample (Fig. 4C). The *Veillonella* ASV5 amplicon from our cohort was closely related to the *Veillonella dispar* amplicon from the Holgerson et al. cohort (Additional file 1: Figure S14B).

### Early-life bacterial communities are associated with concurrent salivary pH, future *S. mutans* prevalence, and primary teeth count

**Fig. 5:**
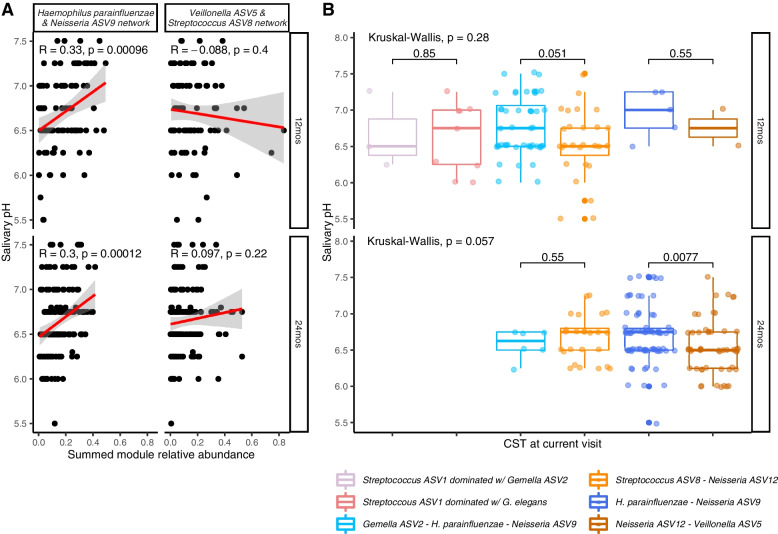
Network modules from weighted co-occurrence network (WCN) graphs and community state types (CST) from community state typing are associated with salivary pH among incident and pre-incident saliva samples from an incidence density-matched nested case-control study selected form the Center for Oral Health Research in Appalachia 2 cohort study. **A** The protective *H. parainfluenzae* and *Neisseria ASV9* network is positively correlated with salivary pH at 12 and 24 months (*n* = 95 at 12 months, with *n* = 71 missing or outlier salivary pH data, *n* = 162 at 24 months with *n* = 10 missing salivary pH data). **B** Salivary pH is also lower in saliva samples that were assigned to the *Neisseria ASV12-Veillonella ASV5* CST when compared to saliva samples assigned to the *Haemophilus parainfluenzae–Neisseria ASV9* CST (*n* = 94 at 12 months, with *n* = 71 missing or outlier salivary pH data and *n* = 1 with unassigned CST, *n* = 162 at 24 months, with *n* = 10 missing salivary pH data)

We tested if bacterial communities from our unsupervised clustering associated with etiologically relevant variables in our cohort. Although salivary pH did not differ between cases and controls at the 12- and 24-month visit (Additional file 1: Figure S15), abundance of the *H. parainfluenzae-Neisseria* ASV9 network module was correlated with increasing salivary pH (12-month rho = 0.33, *P* value < 0.001; 24-month rho = 0.30; *P* value = < 0.001; Fig. 5A). Mean salivary pH was also higher in samples in CSTs characterized by *H. parainfluenzae* and *Neisseria* ASV9 (12-month mean: 6.78; 24-month mean 6.71) than in those characterized by *Streptococcus* ASV8*, Neisseria* ASV12 and *Veillonella* ASV5 (12-month: 6.54, Wilcoxon *P* value = 0.05; 24-month 6.55, Wilcoxon *P* value = 0.01; Fig. 5B).

**Fig. 6:**
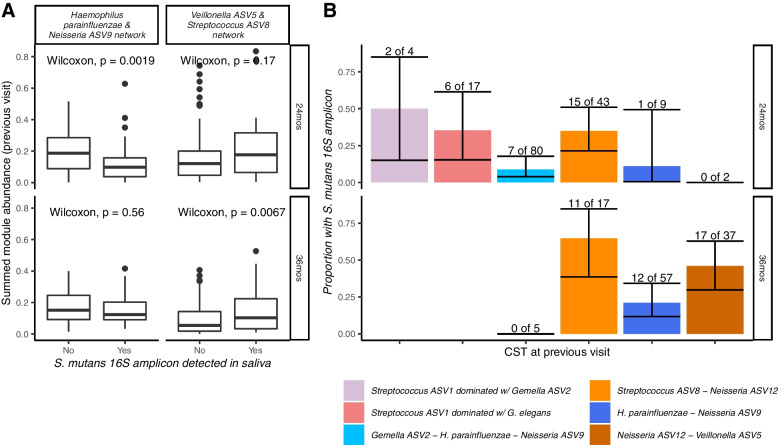
Network modules from weighted co-occurrence network (WCN) graphs and community state types (CST) from community state typing are associated with future detection of Streptococcus mutans among incident and pre-incident saliva samples from an incidence density-matched nested case-control study selected form the Center for Oral Health Research in Appalachia 2 cohort study. **A** Individuals who have *Streptococcus mutans* detected at 24 months have higher abundances of the protective *H. parainfluenzae* and *Neisseria ASV9* network at 12 months (*n* = 157 with both 12- and 24-month clean 16S amplicon data, *n* = 116 with both 24- and 36-month clean 16S amplicon data). **B** Individuals with 12-month and 24-month CSTs characterized by *Haemophilus parainfluenzae* and *Neisseria ASV9* were less likely to have *S. mutans* detected in their 24- and 36-month saliva samples respectively (*n* = s155 with both 12- and 24-month clean 16S amplicon data and *n* = 2 with missing CST at 12 months, *n* = 116 with both 24- and 36-month clean 16S amplicon data)

Children who acquired *S. mutans* by their next visit had lower abundances of the *Haemophilus parainfluenzae-Neisseria* ASV9 network and higher abundances of the *Veillonella* ASV5*-Streptococcus* ASV8 network than children who did not go on to have *S. mutans* (Fig. 6A). Children assigned to CSTs characterized by *Streptococcus* ASV8, *Neisseria* ASV12, and *Veillonella* ASV5 at the 12- and 24-month visits were more likely to have *S. mutans* detected at their next visit than children with communities characterized by *Haemophilus parainfluenzae* and *Neisseria* ASV9 (percent with *S. mutans* at 24-months: 35% vs 9%, Fisher’s exact *P* value < 0.01; at 36 months: 44% vs 21%, Fisher’s exact *P* value < 0.01, Fig. 6B).

The average number of primary teeth present was higher in children assigned to CSTs from later ages. The relative abundance of the *Streptococcus* ASV1-*Neisseria* ASV12 network, which included both *S. mutans* and *Streptococcus sanguinis,* correlated with the number of primary teeth present at the 12- and 24-month visits. This was not true for the protective *H. parainfluenzae-Neisseria* ASV9 network (Additional file 1: Figure S16). For children from Pennsylvania, the approximate age at first tooth emergence was available but was not associated with CST nor network modules.

### Whole-genome shotgun metagenomics of 15 incident case samples and matched controls revealed significant differences in taxa and KEGG ortholog abundances between incident case- and control-samples

**Fig. 7:**
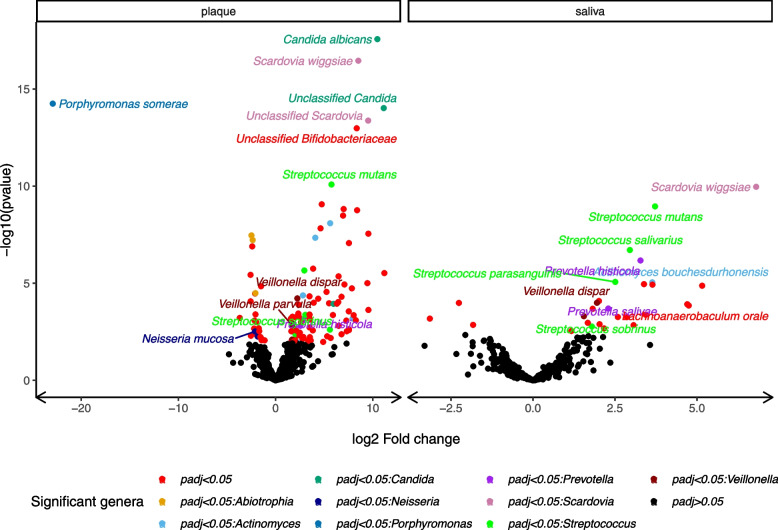
Shotgun-sequenced incident visit plaque and saliva sample exhibited significantly different abundances of taxa among 15 early childhood caries cases and 15 incidence density-matched controls selected from the Center for Oral Health Research in Appalachia 2 study Volcano plots showing the −log10 pvalue and log2 fold change between cases and controls for taxa in plaque and saliva samples from the visit of case diagnosis. Points are colored black if Benjamini-Hochberg *P* value > 0.05 and by genus if the adjusted *P* value < 0.05

We tested for differences in the community composition and functional potential of cases and controls using saliva and plaque samples from the visit of case ECC diagnosis for 15 cases and 15 matched controls. Among others, *Scardovia wiggsiae, Prevotella histicola, Veillonella dispar, Streptococcus mutans and Streptococcus salivarius* were more abundant in case than matched control saliva and plaque samples at the time of diagnosis (Fig. 7; Additional file 13). *Prevotella salivae* was more abundant in case than matched control saliva but not plaque samples (Benjamini-Hochberg *P*_BH_ value < 0.05). The fungal genus *Candida* was only present in case plaque samples.

**Fig. 8:**
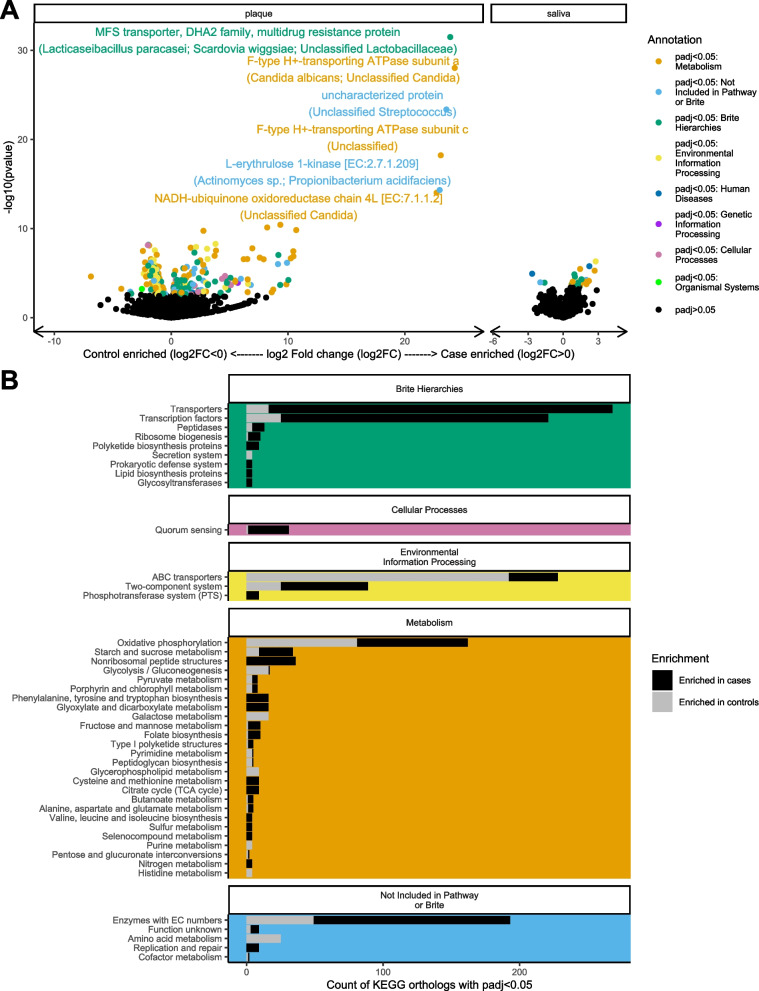
Incident-visit plaque and saliva samples exhibited significantly different abundances of KEGG ortholog groups among 15 early childhood caries cases and 15 incidence density-matched controls selected from the Center for Oral Health Research in Appalachia 2 study. **A** Volcano plots showing the −log10 pvalue and log2 fold change between cases and controls of KEGG orthologs in plaque and saliva samples. Points are colored black if Benjamini-Hochberg *P* value > 0.05 and by the first top-level KEGG annotation from the KEGG hierarchy of the KEGG ortholog if the adjusted *P* value < 0.05. The top 6 most significant KEGG orthologs are annotated with the name of the KEGG ortholog and the taxa in which that KEGG ortholog was found in our sample. **B** Count of KEGG orthologs with Benjamini-Hochberg adjusted *P* value < 0.05 by 3rd level KEGG annotation (*y*-axis), faceted by top level KEGG annotation (colors same as in 3A)

Cases and controls differed in the abundance of gene orthologs (Fig. 8). Associations with case status were stronger in plaque than saliva. Gene orthologs related to antibiotic production and resistance were more abundant in case plaque, including a major facilitator superfamily multidrug resistance transporter (*P*_BH_ value = 1.9 × 10^−28^) and lantibiotic transport system permease protein (*P*_BH_ value = 6.0 × 10^−6^) (Additional file 14). The oxidative phosphorylation KEGG pathway was enriched in case plaque (*P*_BH_ value = 1.2 × 10^−8^), while the ABC transporter pathway was depleted (*P*_BH_ value = 3.6 × 10^−3^, Additional file 15). All the case-associated gene orthologs annotating to oxidative phosphorylation were found only in *Candida* (Additional file 16 and Additional file 1: Figure S17).

## Discussion

Results of our analysis of 99 ECC cases and 90 incidence density-matched children supports the ecologic hypothesis for ECC. We showed that bacteriome-wide information classified future ECC status before reliable detection of salivary *S. mutans*. We expanded on previous work by identifying replicable groups of co-occurring bacteria, which may represent true ecological interactions. We showed that these groups associate with concurrent salivary pH, future *S. mutans* acquisition and future ECC diagnosis, suggesting an ecological succession to cariogenesis. By incorporating shotgun metagenomic sequencing data, we identified functional mechanisms for ecological interactions between bacteria, including pathways related to antibiotic production and resistance. Together, these observations suggest early-life bacterial interactions during a susceptible life period can predispose individuals to ECC.

Our findings on salivary bacteriome assembly and association with ECC fit within the previous literature. We observed a well-documented succession from *Streptococcus-*dominated, low-diversity communities to more diverse communities by 24 months of age with stabilization thereafter [11, 12, 24, 25]. As in cross-sectional dental research, we found an association between *S. mutans* and ECC at the time of ECC diagnosis [8]. Like previous prospective studies of ECC, we found evidence for an association between early life salivary bacteriome composition and future ECC [14, 15]. We were able to distinguish ECC cases from controls more accurately and at an earlier age than reported by Dashper et al., while the AUC-ROC for our 12-month random forest (0.78) is close to that of Grier et al. (0.71) [14, 15]. While *S. mutans*, *S. sobrinus*, and *Scardovia wiggsiae* were elevated in cases at diagnosis, we found that the salivary bacteriome could prospectively predict ECC as early as 12 months, before reliable detection of these risk taxa*.* This supports a time-dependent interpretation of the ecological hypothesis, in which dysbiosis in the oral microbial community precedes salivary *S. mutans* detection, a marker of late-stage cariogenesis. Our findings highlight the first 2 years of life as a susceptible period for assembly of a cariogenic oral microbial community*.*

Unlike most previous work, we identified specific and reproducible ECC-associated bacterial communities using unsupervised clustering techniques. These unsupervised techniques better encapsulate the ecological hypothesis than diversity metrics, which may be too coarse to summarize finer level differences in communities [22]. In our cohort, alpha diversity was weakly and inconsistently associated with future ECC status, echoing previous mixed findings [17–21]. In contrast, groups of taxa from unsupervised clustering techniques were strongly and prospectively associated with ECC: a *Haemophilus parainfluenzae*, *Neisseria*, and *Fusobacterium periodonticum* community was depleted in cases while a *Prevotella*, *Streptococcus*, and *Veillonella* community was more abundant. These communities were distinguished by genetically distinct sequence variants of *Neisseria*, *Veillonella*, and *Fusobacterium*. These bacterial communities were consistent across clustering methods, reproducible in an external cohort [12], and in line with previous work on co-occurrence patterns in oral bacterial communities [26–28].

As our analysis is observational, we can only suggest possible biological explanations for these observed bacterial communities based on previous studies in the literature. These communities could result from habitat filtering by diet, wherein organisms co-occur due to similar nutrient preferences [29]. However, they could also be the result of both cooperative and antagonist ecological interactions, including metabolic exchanges, coaggregation, and interference competition. *Streptococcus* and *Veillonella* species are known to exchange lactic acid and exhibit transcriptional regulation under coaggregation [30, 31]. Fusobacteria are known to play crucial roles in coaggregation with strain-specific impacts on biofilm formation [32–34]. *Streptococcus* [35] and *Neisseria* [36–38] are known to engage in interference competition to outcompete related species, including through the production of bacteriocins such as lantibiotics. Notably, our analysis of shotgun metagenomic sequences identified case-enrichment for gene orthologs for bacteriocin exporters [39] and lantibiotic production [40]. Cariogenic species such as *Streptococcus mutans* are known to use bacteriocins to outcompete other streptococci [35]. Thus, the groups of ECC-associated taxa identified from our unsupervised clustering may reflect ecological interactions, including both intergenera cooperations and intragenera competitions. Future experimental work is necessary to investigate these possibilities.

We also tested how these bacterial communities were associated with etiologically relevant variables. The protective *Haemophilus parainfluenzae*, *Neisseria*, and *Fusobacterium periodonticum* network was correlated with salivary pH and inversely associated with future *S. mutans* detection. In a recent in vitro study, *Neisseria* was positively correlated with salivary pH [41]. This community may therefore be protective by buffering against increases in acidity and subsequent *Streptococcus mutans* colonization. Such a capacity would likely be influenced by host diet and oral hygiene, known etiologic factors in dental decay. In sensitivity analyses, the prospective association between the protective community and ECC diagnosis remained even after adjusting for oral hygiene and diet variables. However, diet is complicated to measure and further investigation is necessary. Future research should investigate relationships between the early-life microbial community, diet, and ECC.

The sample type for assessment of the oral microbiome is an important consideration. We performed 16S rRNA gene sequencing on longitudinal saliva samples, and shotgun metagenomic sequencing on a subsample of cross-sectional plaque and saliva samples. Saliva washes over many oral surfaces with different microbial communities [7, 42–44]. Therefore, differences in bacterial composition of saliva may reflect differences in the bacterial abundance of oral surfaces. Consequently, the co-occurrence patterns we identified may reflect niche-sharing of oral surfaces rather than cooperation between taxa. Although the protective and cariogenic communities we identified were not associated with primary tooth count, we cannot conclusively rule out this explanation. Functional and taxonomic differences were larger in shotgun metagenomic sequenced plaque samples than in saliva samples, and some caries-associated taxa, including *Candida albicans,* were only identified in plaque samples. This may reflect true etiologic differences as plaque, not saliva, is the most proximate tissue in cariogenesis. However, the shotgun metagenomic sequenced saliva samples in our analysis had lower microbial read counts than the plaque samples post-processing, as saliva samples had higher amounts of human DNA, which is expected given that we did not chemically deplete host reads [45]. This could also decrease the power for detecting differences when using saliva samples, especially among low-abundance taxa or functions. Additionally, plaque is more difficult to collect from edentulous children, has a low biomass, and is unlikely to be used as a prognostic marker in a clinical setting. Thus, the predictive power of the early-life salivary microbiome demonstrated in our analysis is of practical, clinical interest. While having both saliva and plaque samples in the shotgun metagenomic sequencing subsample is a strength, our analysis is limited by not including longitudinal plaque samples and by the small sample sizes of this analysis.

Our analysis has several other limitations. The V4 region of the 16S rRNA gene is limited in ability to resolve fine-level taxonomic differences. This could affect the identification and measurement of *Streptococcus* amplicons in our dataset. We validated the identity of *Streptococcus* amplicons using BLAST and shotgun metagenomic data, but nondifferential exposure misclassification of *S. mutans* prevalence is possible. The 16S rRNA gene also does not measure virus, eukaryotes, or interspecies functional variation. Both 16S rRNA gene and shotgun metagenomic sequencing data is inherently compositional. We instituted transformations to address compositionality but did not have absolute abundance data. While we validated the unsupervised clustering methods in an external cohort, we did not have a validation dataset for the supervised random forest.

Our study design is observational, so causality cannot be conclusively proved. However, our exposure measurements precede our outcome, fulfilling a key causal requirement. Our study population was primarily children of European descent from northern and north central Appalachia. Although some of the unsupervised clusters from our cohort were replicable in the Swedish Holgerson et al. cohort, microbial communities can differ by geography, race, and ethnicity. Thus, the generalizability of our findings may be limited. Further studies in additional populations, incorporating shotgun metagenomic sequencing, quantification of absolute microbial loads, and site-specific measures of oral bacterial communities are warranted.

## Conclusions

We found that the early-life salivary microbiome associated with risk of ECC before *S. mutans* could be detected, supporting a time-dependent interpretation of the ecological hypothesis. Our analysis is strengthened by a longitudinal design, balanced case-control ratios, incorporation of both amplicon and shotgun metagenomic sequencing, and replication analyses. Our observations on the suitability of diversity measures vs other clustering techniques to detect fine scale differences are applicable in other microbial contexts. Our findings on ecological succession and bacterial interactions in early life may also be generalizable to other systems of microbiome development. Overall, our analyses support a developmental interpretation of the ecological hypothesis and raise the possibility that ecological interactions and successions in early life, in addition to etiologic risk factors such as diet and oral hygiene, may predispose children to ECC.

## Methods

### Study cohort

We used data from the Center for Oral Health Research in Appalachia 2 study (COHRA2) [46]. COHRA2 recruited White, pregnant women between 2011 and 2015 from Pennsylvania and West Virginia. Healthy women who were in the 12th to 29th week of pregnancy, of European descent, over 18 years of old, fluent in English, and with a singleton pregnancy were eligible for inclusion. Women and their babies were followed longitudinally through the early years of the baby’s life. Women were excluded if they had tuberculosis, were immunocompromised, thought they might soon leave the general regions of West Virginia or southwestern Pennsylvania, or did not have a reliable telephone contact. Mother-child pairs also were excluded from the study if the child was delivered before the 35th week of pregnancy or if the mother or child developed a serious medical condition.

Participants completed in-person visits when the child was 2 months and 12 months old, then yearly thereafter. Mother-child pairs from the Pennsylvania site had additional in-person visits at birth and when the child’s first primary tooth erupted. At in-person visits mothers and children underwent a comprehensive dental assessment by a trained and calibrated dental professionals (training and calibration described in detail in Neiswanger et al. [46]); participants were asked not to eat or drink for 2 hours prior to the examination. The examination included caries assessment via the PhenX Toolkit Dental Caries Experience Prevalence Protocol (http://www.phenxtoolkit.org/, protocol number 080300) which allows for the decayed, missing, and filled tooth count to be calculated either including or excluding white spots. The dental examination also included collection of microbial samples from saliva, plaque, and gingival swabs using OMNIgene Discover kits (OM-501 or 505 DNA Genotek); only saliva and plaque samples were used in this analysis. Saliva was collected via swabs for children too young to spit into a collection tube and via spitting otherwise. Pooled plaque samples were taken with a Stimudent or curette from three intact tooth surfaces (in UNS/FDI notation: 8-buccal/51-buccal, 24-buccal/71-buccal, 31-occlusal/84-occusal or nearby surfaces if these were not intact). Plaque was also taken from tooth surfaces with untreated dental lesions. Salivary pH was also measured at visits where the child was old enough to spit (most by 12 months, all by 24 months) using a pH strip.

A 30–45-min telephone interview was administered to the mothers at approximately 6-month intervals to capture sociodemographic and behavioral data. These interviews included questions about oral hygiene and approximate frequency of child consumption of specific foods and beverages.

### Sampling and case definition

For this analysis we selected 99 children who had any dental lesions, including white spots (d1mft), at or prior to the 60-month visit in the 2019 data freeze of the COHRA2 cohort as early childhood caries (ECC) cases. The visit in which a child was first identified as having a dental lesion or white spot was the incident-visit for that child. We then selected a similar number of children who were free of dental lesions and white spots at the same visit as the cases to serve as incidence-density sampled controls (*n* = 90). Incidence density sampling does not preclude the reselection of a control as a case at later time points; controls can also be selected as controls for multiple cases (Fig. 1) [47]. In this analysis, one control was later selected as a case and one control was selected as a control twice (*n* = 92 control records). Duplicate records of the case/control and control/control children were not used in the supervised random forest: the case/control was only included as a case and the control/control was only included as a control once. In both unsupervised clustering techniques, we did not include duplicate records from these individuals when performing initial clustering or in the supervised random forests but did include them when graphing and testing associations between identified clusters and variables of interest (i.e., in Table 1, Figs. 1, 2, and 3). The number of total unique individuals in the analysis was 189, with 191 unique person-records (Additional file 5).

All available saliva samples from cases and controls, up to and including the incident-visit saliva sample, were pulled for 16S rRNA amplicon sequencing (Fig. 1). Note that selected individuals occasionally missed visits, did not have a saliva sample available, or had a saliva sample which failed 16S amplicon quality control (Additional file 5). Additionally, we randomly selected a subcohort of 15 cases presenting with enamel lesions at or after the 36-month visit and 15 corresponding controls. Plaque and saliva samples from the visit of case diagnosis for these 30 individuals were submitted for shotgun metagenomic sequencing.

### Laboratory and bioinformatics pipeline for 16S rRNA amplicon metagenomic sequencing

Bacterial DNA was extracted from aliquots of saliva. Library preparation and sequencing of the 16S rRNA V4 amplicon was performed by the Michigan Microbial Systems Molecular Biology Laboratory using previously validated protocols [48]. DNA extraction was performed using the Eppedorf EpMotion liquid handling system following the Qiagen MagAttract PowerMicrobiome kit protocol. The V4 variable region was amplified from extracted DNA using barcoded dual-index primers and sequenced on the Illumnia MiSeq platform using the MiSeq Reagent Kit V2 500 cycles. Each plate of samples was submitted with a positive mock community control, a DNA extraction kit control, and a negative water control (Additional file 2: Figures S3–4). Reads were processed to amplicon sequence variants (ASVs) using DADA2 (version 1.14.1) [49] and the Human Oral Microbiome Database (HOMD) version 15.2 [50]. To identify contaminants, we used the R package decontam (version 1.8.0) [51]. We filtered out samples with less than 1000 reads (*n* = 6 samples lost). Diversity metrics were calculated using the estimate_richness function from the R package phyloseq all ASVs. However, to limit the number of features used in supervised and unsupervised learning, we instituted a prevalence-abundance ASV filter. ASVs which were present in less than 5% of all samples *and* which represented less than 5% of all sequences in the samples in which they were present were excluded from the analytic subset for supervised random forest and unsupervised clustering techniques (*m* = 273 ASVs in analytic subset). ASVs were not collapsed at the genus or species level.

### Random forest

We used the 12- and 24-month visits as inputs for the random forest as these visits preserved a large subset of pre-incident samples. Only pre-incident cases and matched controls were used in the random forest: individuals with incident-visit saliva samples were excluded (6 individuals with available samples who were identified as cases or controls at the 12-month visit and 37 individuals identified at the 24-month visit were excluded, total sample size of *n* = 158 and *n* = 132). Hellinger transformed ASV counts from the 273 ASVs in our analysis subset were used in the random forest. Using the train function in the R package caret [52], we ran 5 repeats of 10-fold cross validated random forest machine algorithms with 500 trees. We allowed the mtry parameter (number of parameters randomly sampled as candidates at each tree split) to be tuned from a choice of 2, 136, or 271 using the receiver operating characteristic curve; for both the 12-month and 24-month all taxa random forest an mtry parameter of 2 was selected. Area under the receiver operating curve and other evaluation statistics were calculated using the R package MLeval [53].

### Dirichlet multinomial community state typing

We used the R package DirichletMultinomial to cluster samples into community state types (CSTs) using Dirichlet multinomial mixture models [54]. We fit ten Dirichlet multinomial models, using as input the count matrix of the 855 samples by 273 ASVs in the analytic subset and varying the number of Dirichlet components (i.e., CSTs) from 1 to 10. We calculated the Laplace measure of fit for each model and plotted against *k,* identifying *k* = 6 as the best model. We varied *k =* {4, 5} as a sensitivity analysis. Samples were assigned to the single *k* CST for which they had the highest posterior probability of membership; if a sample assigned to no CST at a posterior probability > 80%, the sample was not assigned to any CST.

### Weighted co-occurrence networks

We used the R package WGCNA to build a signed weighted network of ASVs using the Hellinger-transformed count matrix of 855 samples and 273 ASVs [55]. As a sensitivity analysis, we used the center-log ratio transformed count matrix. The soft thresholding power of the signed network was selected to maximize the R^2 of the model fit while preserving the mean connectivity of the network using the pickSoftThreshold function in WCGNA. We used a dynamic tree cut and the cutreeDynamic function in WCGNA to identify network modules or clusters using a minimum module size of 5 and a deep split value of 4, with the aim of producing more fine-grained clusters. Intramodular connectivity statistics were calculated for each ASV using the intramodularConnectivity function. Finally, per-sample module relative abundances were calculated by summing the relative abundances of all ASVs belonging to the same module.

### Replication cohort

We performed the exact same bioinformatics and analytic pipeline on publicly available V3-–V4 16S rRNA gene data from the Holgerson cohort (PRJEB35824) [12], as we did to the COHRA2 samples. This cohort was also composed of sequential salivary samples from similarly aged children, the prevalence of ECC was 10% by 60 months of age. The laboratory methods for these samples are described in Holgerson et al. [12]*.* All the bioinformatics parameters and steps were the same as described above, with the exception that decontam was not used to identify potential contaminants as the publicly available data did not include DNA quantification data. Since we could not obtain access to any metadata characteristics of these samples, including ECC status, the random forest models could not be run. For visualization purposes, the two matching networks shown in Fig. 4C were filtered to only edges with a weight > 0.03. The full, unfiltered network images are shown in Additional file 1. To compare the relatedness of the amplicon sequence variants assigned to various network modules across the COHRA2 and Holgerson cohort, we performed multiple sequence alignment of the amplicons using the R packages msa, using the ClustalW algorithim [56]). We computed pairwise distances from the DNA sequences using the r function dist.dml from the r package phangorn [57], using the JC69 model. We created a neighbor joining tree using the phangorn function NJ, then fit a generalized time-reversible with gamma rate maximum likelihood tree using the neighbor joining tree as a starting point. We obtained 100 bootstrap values for the tree using bootstrap.pml and plotted the tree using ggtree [58] and collapsed branches present in < 50 of the bootstrapped trees.

### Statistical analyses

We investigated differences between cases and controls in salivary pH using boxplots and Wilcoxon’s test. At 12 months, *n* = 97 children had both clean 16S amplicon data and salivary pH measurements and at 24 months, *n* = 168 children did. As we could not exclude the possibility of technical errors associated with measuring salivary pH using a test strip, we excluded from consideration samples with a salivary pH < 5.5 (*n* = 2) or > 8 (*n* = 6), leaving *n* = 95 and *n* = 162. We investigated associations between salivary pH and summed module abundance using Pearson’s correlation coefficient and scatter plots. We investigated associations between salivary pH and CST using Fisher’s exact test and bar graphs (additional *n* = 1 child excluded at 12 months due to unassigned CST). Among *n* = 157 with both 12- and 24-month 16S amplicon data and *n* = 116 with both 24- and 36-month amplicon data, we investigated differences in summed module abundance by next-visit *Streptococcus mutans* detection using boxplots and Wilcoxon’s rank sum test and differences in proportions of children with CSTs by next-visit *Streptococcus mutans* detection using bar plots and Fisher’s exact test (additional *n* = 2 children with unassigned CST excluded). We used logistic regression to test for associations between summary metrics from unsupervised clustering and ECC separately at the 12- and 24-month visits while controlling for potential confounders identified from literature review and directed acyclic graphic. For each time point only pre-incident cases and controls were included in the regression, i.e., cases diagnosed at 12 months of age and age-matched controls were not included in the 12-month regression models. This ensures that the regression models test for prospective associations between current salivary bacteriome and future ECC diagnosis. To test for associations between CST and future ECC diagnosis, ECC status was used as the outcome and CST assignment was included as a categorical predictor. To test for associations between network modules and future ECC diagnosis, ECC status was used as the outcome and relative abundance of the network module was included as a continuous predictor ranging from 0 to 100. This allows the exponentiated coefficient to be interpreted as the odds ratio for a 1 percentage point increase in network module abundance. In adjusted models we included the following covariates: binary indicator for child being currently breastfed at the visit, binary indicator for maternal report of child antibiotic use within 3 months of visit, count of emerged primary teeth, binary indicator for birth delivery mode, binary indicator for maternal education greater than high school, and categorical variable for visit of case diagnosis/control matching. As a sensitivity analysis we also controlled for tooth brushing/wiping (none, yes without toothpaste and yes with toothpaste) and past week juice consumption frequency (never or once, a few days, every day, several times per day).

### Laboratory and bioinformatics pipeline for shotgun metagenomic sequencing

DNA was extracted from plaque and saliva samples using the Zymobiomics miniprep kit according to the manufacturer’s instructions. Isolated DNA was quantified by Qubit. DNA libraries were prepared using the Illumina Nextera XT library preparation kit according to the manufacturer’s protocol. Library quantity and quality was assessed with Qubit (ThermoFisher) and Tapestation (Agilent Technologies, CA, USA). Libraries were then sequenced on Illumina HiSeq platform 2 × 150 bp. Quality filtering and adapter trimming were performed using Trimmomatic and the Nextera PE adapters. Host DNA was removed using bowtie2 and the GRCh38 index. Trimmed, cleaned and decontaminated reads were processed through both the Humann3 short-read profiling pipeline [59] and the SqueezeMeta assembly-based pipeline (version 1.4.0) [60]. Plaque and saliva samples were run separately through the assembly pipeline. Briefly, assembly was done using Megahit, ORFs were predicted using Prodigal, and similarity searches against GenBank, eggnog and KEGG were conducted using Diamond. Read mapping against contigs was performed using Bowtie2. Binning was done using MaxBin2 and Metabat2 and bins were combined using DAS Tool. To test for differential abundance of KEGG orthologs and taxa abundance estimated from contigs, we used DESeq2, first filtering out KEGG or taxa with fewer than 500 reads from the testing subset. We tested for enrichment in KEGG pathways using gene set enrichment analysis and the R package fgsea separately on plaque and saliva samples. We used the package SQMTools to extract functional and taxonomic subsets of interest, such as the KEGG orthologs which annotated to oxidative phosphorylation. To test correlations between 16S rRNA gene amplicon sequence variants and abundances of taxa from whole genome sequencing, we used a partial spearman correlation while controlling for incident visit and case status.

## Supplementary Information

**Additional file 1: **Supplementary methods (text) and **Supplementary** **figures**.

**Additional file 2: Supplementary Table.** Distribution of key characteristics among non-case children in the Center for Oral Health Research in Appalachia 2 cohort and among the children sampled into the nested case-control analysis set as controls.

**Additional file 3: Supplementary Table.** Additional case severity statistics by 12- and 24-month microbial community state type in the COHRA2 incidence-density sampled case-control subset.

**Additional file 4: Supplementary Table.** Additional case severity statistics at the individual level.

**Additional file 5: Supplementary Table.** Detailed sample loss by visit due to missing metadata, missing saliva samples, and quality filtering of 16S rRNA amplicon sequencing data.

**Additional file 6: Supplementary Table.** Demographics of the subset with shotgun metagenomic sequenced plaque and saliva samples.

**Additional file 7: Supplementary Table.** Top 100 best matches from BLAST search of the 16S rRNA amplicon V4 region of *Streptococcus* ASVs of interest in our sample (ASV14, ASV8, and ASV82).

**Additional file 8: Supplementary Table.** Distribution of key microbial features (alpha diversity metrics, weighted co-occurrence networks, community state types, and prevalence/abundance of *a priori* taxa) by case status from saliva samples of visit of case diagnosis and control matching.

**Additional file 9: Supplementary Table.** Distribution of key microbial features (alpha diversity metrics, weighted co-occurrence networks, community state types, and prevalence/abundance of *a priori* taxa) by case status from saliva samples of visits preceding case diagnosis/control matching.

**Additional file 10: Supplementary Table.** ASV membership in the five weighted co-occurrence network modules presented in the main analysis. Each network module is a tab in the file, each row is an ASV belonging to that network module. ASV number, scientific name, module label (top two most abundant taxa in the module + the most central taxa in the module) and measures of total (kTotal), within module (kWithin) and between module (kOut) connectivity/degree (as calculated by the *intramodularConnectivity* function in the WCGNA package), as well as the difference between within and between module connectivity (kDiff) for each ASV.

**Additional file 11: **Rand index, adjusted rand index and adjusted mutual information index for alternate clustering parameters in the community state typing (k=4, 5, or 6 clusters) and weighted co-occurrence networks (Hellinger vs center log ratio transformation).

**Additional file 12: **Food frequency of high-sugar foods and tooth brushing/wiping data by case status and child visit.

**Additional file 13: Supplementary Table.** Results from DeSeq analysis testing for differential taxa abundances between incident visit case and control plaque and saliva samples. All tested taxa included, sorted by adjusted *P* value.

**Additional file 14: Supplementary Table.** Results from DeSeq analysis testing for differential KEGG ortholog abundances between incident visit case and control plaque and saliva samples. All tested KEGG orthologs included, sorted by adjusted *P* value, includes path annotation.

**Additional file 15: Supplementary Table.** Results of gene set enrichment analysis performed using the R package fgsea and the results from DeSeq performed on KEGG orthologs.

**Additional file 16: Supplementary Table.** Taxonomic annotation and log2 Fold Change values for KEGG orthologs annotating to the oxidative phosphorylation pathway.

## Data Availability

The 16S rRNA gene amplicon sequencing data and shotgun metagenomic sequencing data from the COHRA2 study is publicly available at the PRJNA752888 repository. The 16S rRNA gene amplicon sequencing data from the Holgerson et al. replication cohort is publicly available at the PRJEB35824 repository. Phenotype data for the COHRA2 study are available at dbGaP phs001591.v1.p1 upon application. All the code to reproduce the analyses in this paper is available at *https://github.com/blostein/ECCPaper1**.*

## Abbreviations

ECC: Early childhood caries
ASV: Amplicon sequence variant
16S rRNA: 16S ribosomal RNA
CST: Community state type
