# Supplementary material for: Evaluating the ecological hypothesis: early life salivary microbiome assembly predicts dental caries in a longitudinal case-control study
Source: Microbiome. 2022 Dec 26;10:240. doi: 10.1186/s40168-022-01442-5 (PMC9791751; doi:10.1186/s40168-022-01442-5)
Supplement: Supplementary file 2 — Additional file 1: Supplementary methods (text) and Supplementary figures. [file 40168_2022_1442_MOESM1_ESM.pdf]

Testing the ecological hypothesis: Longitudinal case-control study of early childhood caries and salivary microbiome assembly supplementary section

Freida Blostein

2022-11-18

Description of study sample

We used a nested case-control design with incident sampling to sample sequential saliva samples from 99 ECC cases and 90 incidence-visit matched controls from the Center for Oral Health Research in Appalachia 2 cohort (COHRA2), using the 2019 data freeze. Children in COHRA2 are followed from birth until 5 years of age with regularly scheduled study visits in which they both provide oral samples and are examined for dental decay. Children therefore enter the cohort at birth, and all children are at-risk for developing ECC upon entry into the cohort. At each visit in which cases were diagnosed, we selected a similar number of ECC-free controls from the risk-set of all individuals still at risk of ECC, i.e. all those individuals who have not yet been diagnosed with ECC. Incidence density sampling does not preclude the re-selection of a control as a case at later time points; controls can also be selected as controls more than once. In this analysis, one control became a case at a later age, and one control was selected as a control in two different risk-sets.

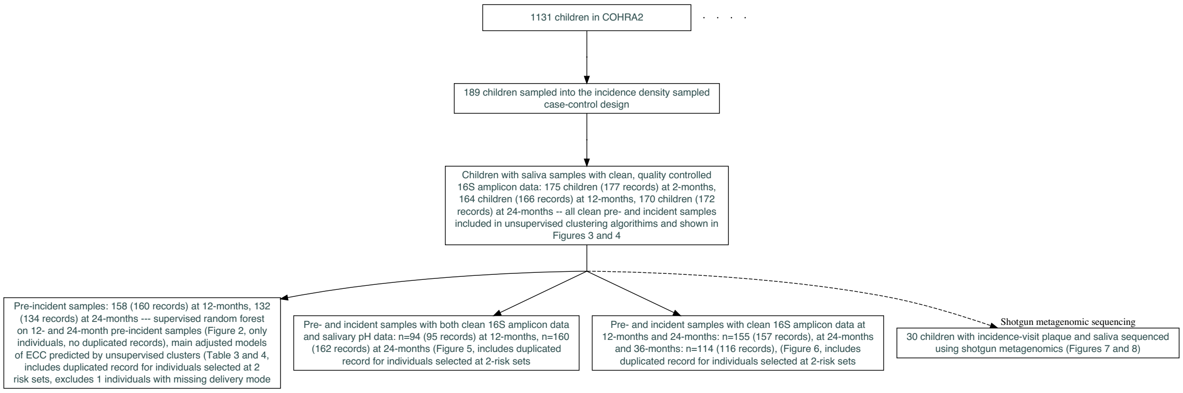

Figure 1: Flow chart of samples included in each analysis

Additional file 2 shows the distribution of some key characteristics in the children sampled as controls into the nested case control cohort and in the overall disease free cohort at the 2-month visit. Additional files 3 and 4 have additional data on case phenotype presentation.

Among the nested case-control sample, cases and controls did not differ in the distribution of race, sex, mode of birth delivery, or site of recruitment. Cases and controls also did not differ in maternal report of child receipt of antibiotics within the 3 months immediately prior to a visit. At each of the 2-, 12-

and 24-month visits, cases and controls had similar numbers of erupted primary teeth. Fewer cases (49%) than controls (63%) were breastfed at the 2-month visit, although this was not a statistically significant difference (P=0.07). The mothers of cases were less likely to have an education beyond a high school degree (33%) than the mothers of controls (63%, P<0.001).

All available saliva samples from the cases and controls from the visit in which they were selected and all preceding visits were pulled for 16S rRNA amplicon sequencing. Selected individuals occasionally missed visits, did not have a saliva sample available, or had a saliva sample which failed 16S amplicon quality control (Additional file 5).

Additionally, we randomly selected a subcohort of 15 cases presenting with enamel lesions at or after the 36-month visit and 15 corresponding controls. Incident-visit plaque and saliva samples from these 30 individuals were submitted for whole genome shotgun metagenomic sequencing. Since only children who developed dental decay at or after the 36-month visit were eligible to be sampled into the shotgun metagenomic sequencing project, these children are not truly representative of the entire nested case-control study. However, the subsample submitted for metagenomic sequencing did not significantly differ from the overall nested case-control sample in the distribution of race, sex, recruitment site, maternal education, maternal report of antibiotics in the 3-months preceeding the 2-, 12- and 24-month visit, breastfeeding at the 2-, 12-, or 24-month visit (see Additional File 6).

16S amplicon bioinformatics

Sequencing

Samples were sequenced over 3 runs (each with 4 plates). Each plate included a positive mock control (ZymoBIOMICS Microbial Community DNA Standard (Zymo Research cat# D6306) and 2 negative controls (extraction control and water control). We processed sequenced reads. Sequencing was done on the Illumina MiSeq platform, using a MiSeq Reagent Kit V2 500 cycles (Illumina cat# MS102-2003), according to the manufacturers instructions with modifications found in the Schloss SOP. Accuprime High Fidelity Taq (Life Technologies cat # 12346094) was used instead of Accuprime Pfx supermix. PCR products are visualized using an E-Gel 96 with SYBR Safe DNA Gel Stain, 2% (Life technologies cat# G7208-02). Libraries are normalized using SequalPrep Normalization Plate Kit (Life technologies cat # A10510-01) following the manufactures protocol for sequential elution. The concentration of the pooled samples is determined using Kapa Biosystems Library Quantification kit for Illumina platforms (KapaBiosystems KK4824). The sizes of the amplicons in the library are determined using the Agilent Bioanalyzer High Sensitivity DNA analysis kit (cat# 5067-4626). The final library consists of equal molar amounts from each of the plates, normalized to the pooled plate at the lowest concentration. Library Preparation for Sequencing and Sequencing Libraries are prepared according to Illumina's protocol for Preparing Libraries for Sequencing on the MiSeq (part# 15039740 Rev. D) PhiX (15%) was added in 16s amplicon sequencing to create diversity. Sequencing reagents are prepared according to the Schloss SOP, custom read 1, read 2 and index primers are added to the reagent cartridge. FASTQ files are generated for paired end reads. DNA was quantified using PicoGreen.

DADA2 processing

Reads were processed to amplicon sequence variants (ASVs) using DADA2 (version 1.14.1) and the Human Oral Microbiome Database (HOMD) version 15.2. After examining quality plots for forward and reverse reads, reads were trimmed at the 240th and 200th nucleotide position respectively, or at the first instance of a quality score <= to 11. The following parameters were used for filtering reads: a maximum of 2 expected errors for both forward and reverse reads and no ambiguous bases.

Errors were learned (independently for each run) using 1 million bases. The forward and reverse reads were denoised and then forward and reverse reads were merged together.

At this point, the runs were merged together, and chimeras were removed using DADA2s removeBimeraDenovo commmand and the consensus method. The mean read number, prior to any sample or taxa filtering and including both positive and negative controls, was 2.1344×10^4, sd=8939. A total of m=3194 ASVs were identified.

Subsequently, reads were assigned taxonomic names to the species level using the assignTaxonomy and the addSpecies commands of DADA2. A modified version of the Human Oral Microbiome Database (HOMD) version 15.2 (modified by adding the exact sequences of the positive control samples) was used.

Sample and taxa filtering

To identify potential contaminant taxa we used the R package decontam, identifying contaminants using both the frequency-based method (PicoGreen quantification used as measure of concentration of amplified DNA), and the prevalence based method; thresholds set at 0.1 and 0.3 respectively. Contaminant identification was performed independently within each batch. Of the original 3194 ASVs, 104 ASVs were identified as contaminants and removed.

Next, we filtered out samples that had less than 1000 reads (Supplemental Figure 2; red line at read limit=1000). Of a total original 864 samples, 6 samples had < 1000, reads, leaving 858 samples. Three of the true samples which passed quality control were missing all metadata and were excluded, leaving a total of 855 in the analysis. After restricting to just the true samples (not controls) with > 1000, 3033 ASVs remained. Calculation of diversity metrics (Shannon index, Chao1) was performed on these ASVs. ASVs were not collapsed to the genus or species level.

Of the original 12 mock communities, 1 had less than 1000 reads; the other mock communities in the same run on other plates amplified and were as expected (Supplemental Figure 3). None of the extraction controls had more than 1000 reads, and one water control did, this water control had <3000 reads of highly common taxa in the analytic subset and likely reflects some cross-contamination.

Finally, for the unsupervised and supervised analysis, we further restricted the ASV set to ASVs. We filtered out ASVs that were both 1) present in less than 5% of the samples and 2) represented less than 5% of the relative abundance in samples in which they were present. This left a total of 273 ASVs.

Supervised random forest

Additional investigation of *Streptococcus* amplicons identity

Species-level identification of *Streptococcus* with the V4 amplicon of the 16S gene is not perfect. Two amplicons identified as *Streptococcus* were important features in the taxa-wide random-forest, but did not identify to the species level. The identity of the amplicon identified as *Streptococcus mutans* is central to our test of the ecological hypothesis. We performed several analyses to improve our confidence in the taxonomic classification of these important *Streptococcus* amplicons.

Streptococcus ASV82, which identified as *S. mutans*, had 100% identity with *Streptococcus mutans* in a BLAST search. 6 of the top 10 top hits were *S mutans.* None of the other 4 top 10 hits were human oral *Streptococcus* species (Additional file 4).

Neither *Streptoccocus ASV8* nor *Streptoccocus ASV14* had a high identity with any *S. mutans* 16S sequences when performing a BLAST search. *Streptococcus ASV8* had 100% identity with *Streptococcus thermophilus and Streptococcus vestibularis ATCC 49124 and Streptococcus salivarius*, *Streptococcus ASV14* with *Streptococcus peroris ATCC 700780 and Streptococcus lactarius.* Neither *Streptococcus ASV8* or *ASV14* had an *S. mutans* BLAST hit in the top 100 best blast hits (Additional file 7).

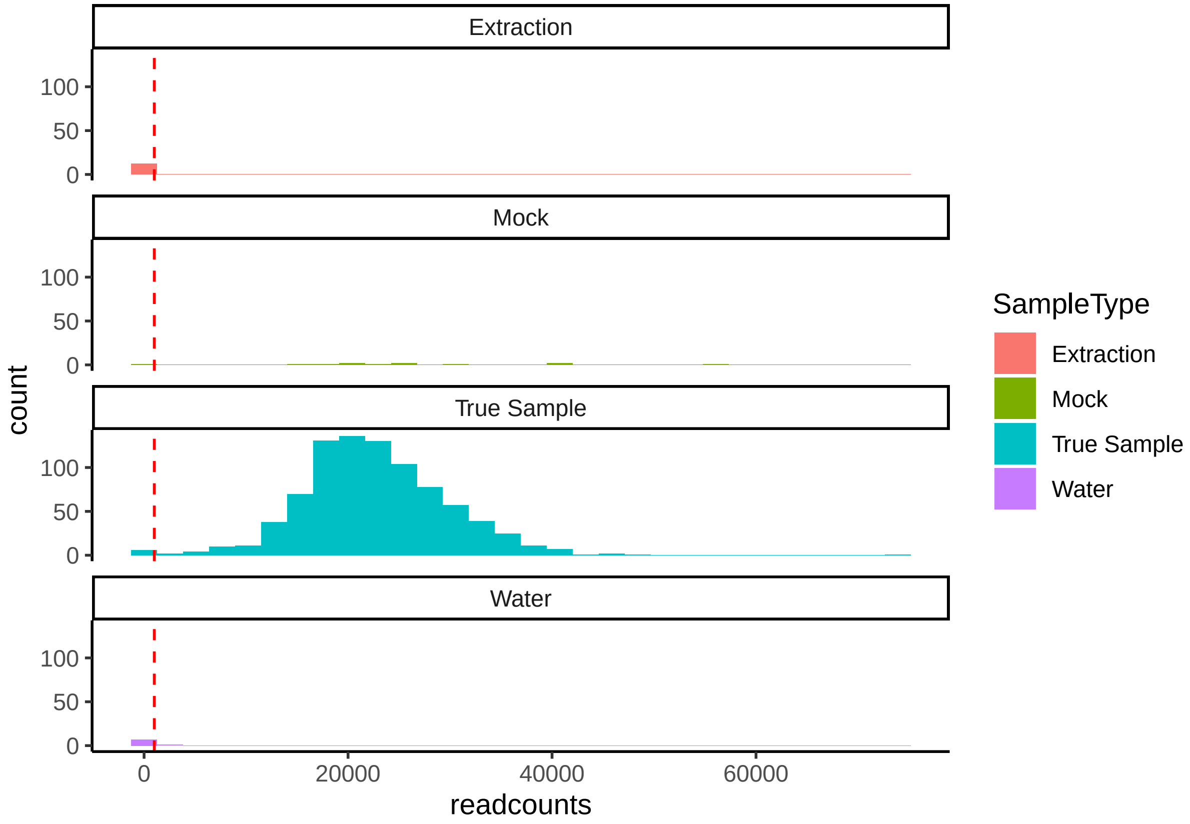

Figure 2: Read count by sample type in true samples and positive and negative controls

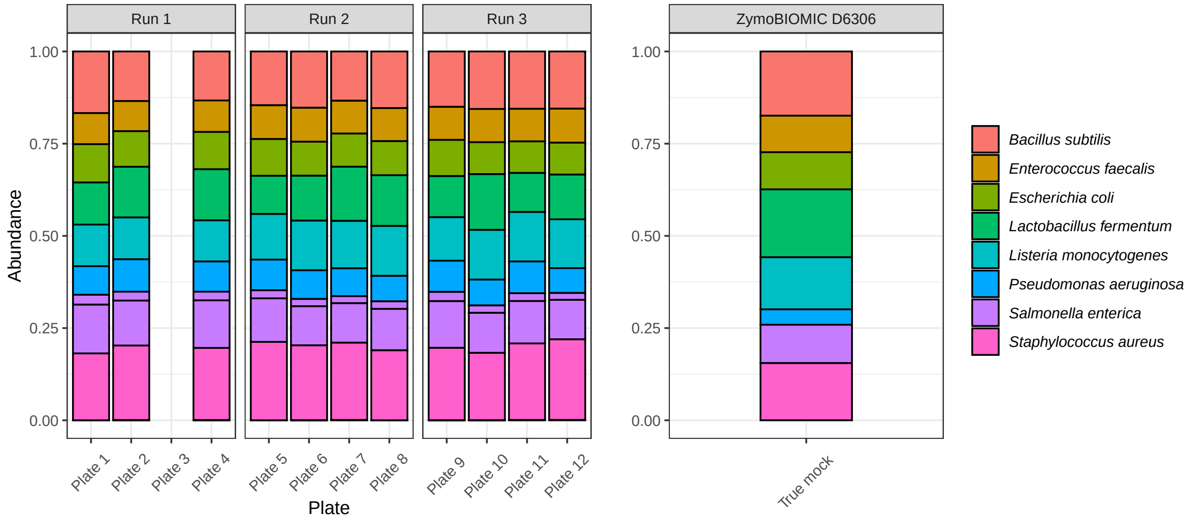

Figure 3: Mock communities by sequencing run as compared to true mock abundance

Additionally, we exploited our subcohort with metagenomically sequenced saliva samples to interrogate the identity of the *Streptococcus* amplicons. Abundance of the 16S amplicon *Streptococcus ASV82* (*S. mutans*) was strongly and significantly correlated with abundances of *S. mutans* in WGS samples when using either Humann3 profile abundances or abundances estimated from an assembly based pipeline, while amplicon abundances of *Streptococcus ASV8* correlated strongly and significantly with *S. salivarius* and *Streptococcus ASV14* with *S. australis* and *S. infantis* (Supplemental Figure 4, Pearson r values significant at P value<0.05 shown as text in the heatmap).

The prevalence and abundance of *S. mutans* in case and control samples from visits prior to case diagnosis are shown in Main table 1, while the prevalence and abundance of *S. mutans* in case and control samples from the visit of case diagnosis are in additional file 8.

Unsupervised clustering techniques

Community state typing: choice of k community state types

Using Dirichlet multinomial community state typing, we identified 6 community state types (also called enterotypes). We chose k=6 community state types as this choice of k produced the best model fit as measured by the LaPlace metric (Supplemental Figure 5). Each sample is assigned to a single community state type based on a posterior probability; any sample which did not assign to a community state type at a posterior probability >80% was labeled unclassified. Community state types were named by the taxa defining their separation. The distribution of community state types in incident visits is in Additional file 8 and in preincident visits is shown in addition file 9.

Supplemental Figure 6 shows the relationship between Shannon diversity and community state type. Each dot is a single sample which has been assigned to a community state type (x-axis), the Shannon index in that sample is on the y-axis Community state type is significantly associated with Shannon diversity. As children age, the dominant community state types progress from those that are *Streptococcus* dominated to more diverse community state types. The progression of community state types in both cases and controls follows this pattern.

Weighted co-occurence network analysis

Using weighted co-occurrence network analysis, we identified 5 network modules of co-occurring ASVs. These networks were named after the top two most abundant taxa in the network, and the most central taxa in the network. The membership of all of the networks is in Additional file 10. To summarize these network modules, we summed the relative abundances of each taxa belonging to the network module compared the trajectories of these abundances over time between cases and controls. Change in the summed relative abundance of the 3 network modules not displayed in Figure 2B are shown in Supplemental Figure 7; the structure of the overall network is shown in Supplemental Figure 8. In Supplemental Figure 8, ASVs are nodes in the network. ASVs are colored by the module to which they were assigned and sized according to their abundance. The shape of the ASV node indicates if that ASV is more abundant in ECC cases (triangles) or controls (squares). Edges with a weight<0.1 have been eliminated from the network visualization for readability, otherwise edge width corresponds to the strength of the correlation between nodes. ASVs of special interest are labeled.

Cases and controls differed in the summed relative abundance of these network modules and the distribution of community state types in both pre-incident (aditional file 9) and incident (Additional file 8) visits.

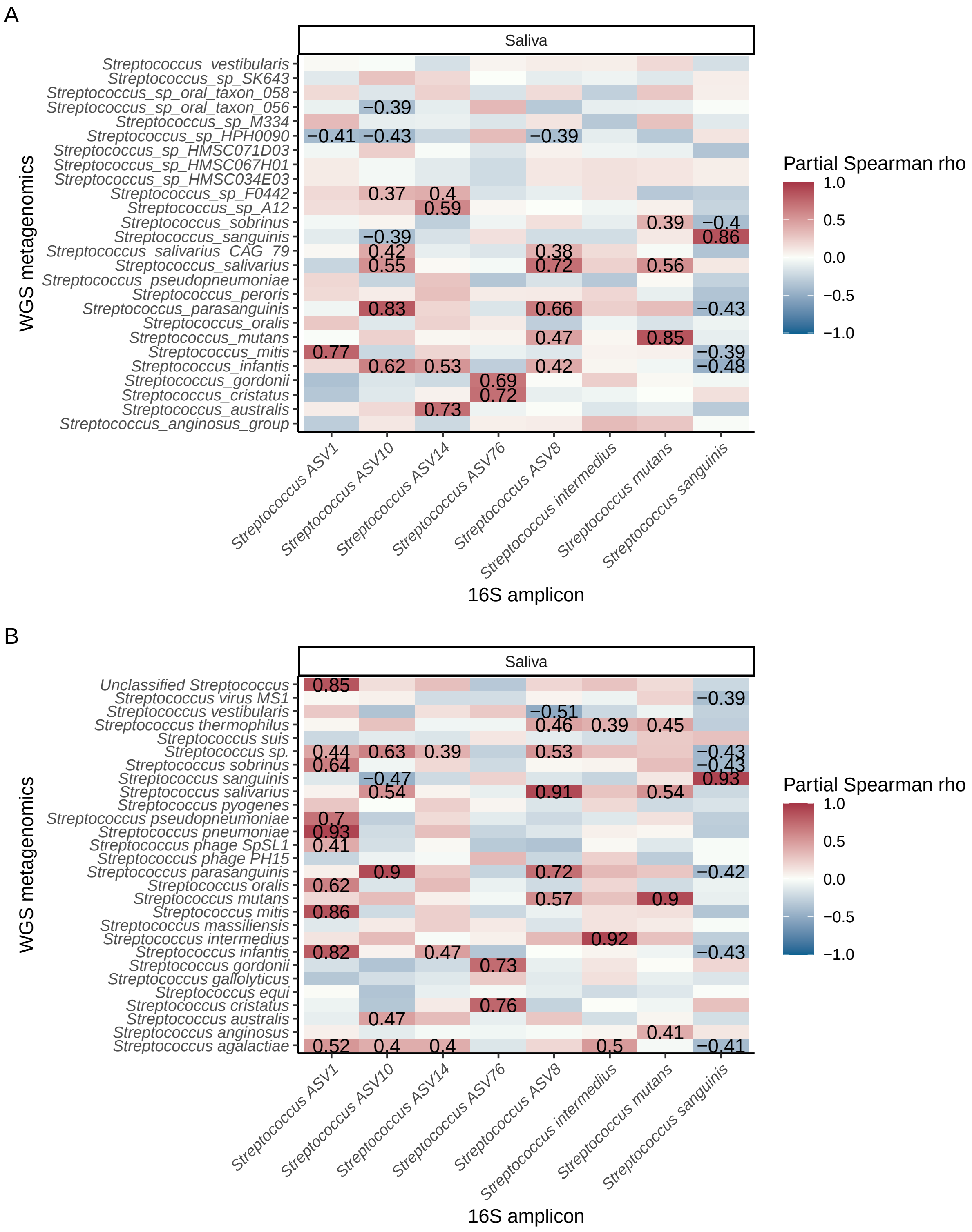

Figure 4: Correlation between Streptococcus amplicons from 16S rRNA gene sequencing and taxa identified from contiguous sequences of whole genome shotgun sequening

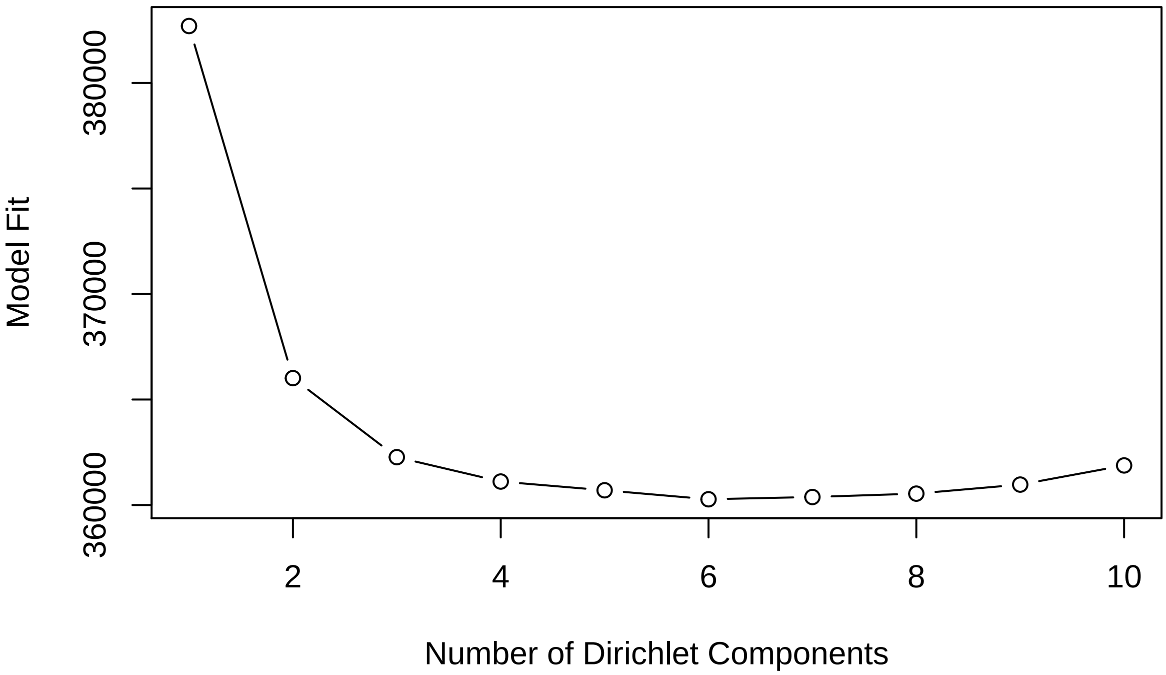

Figure 5: Laplace model fit at k=1:10 community state types

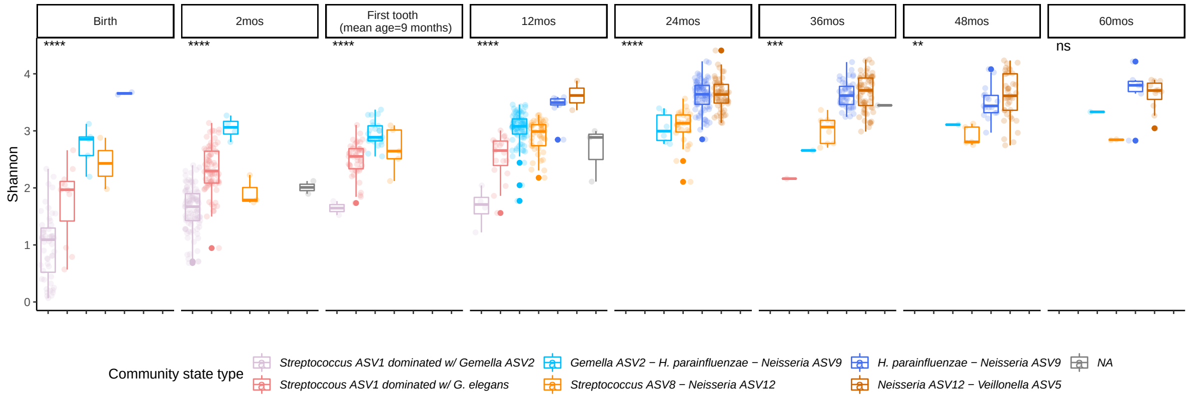

Figure 6: Shannon index in samples by visit and community state type

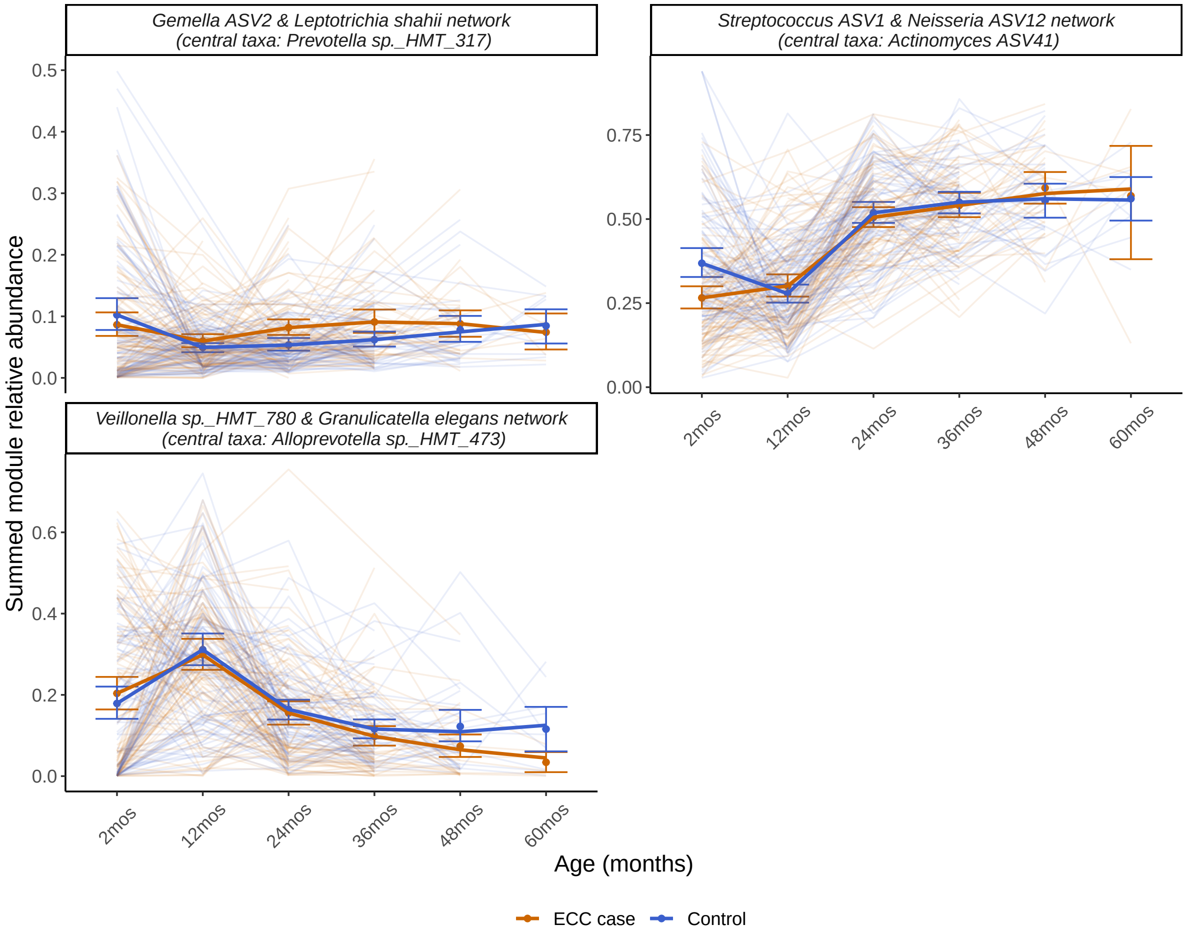

Figure 7: Summed network module relative abundance for the network modules in the COHRA2 cohort not shown in Figure 2 of main paper

Overlap between top ten separation taxa from community state typing and top two most abundant taxa from each network modules

Supplemental Figure 9 shows the overlap between the top two most abundant ASVs in each of the five network modules (ten ASVs) and the top ten most important ASVs for the separation of the community state types. There is significant overlap in these ASVs, with nine of the ten ASVs being the same.

Robustness analysis

For the Dirichlet multinomial community state typing, we varied the choice of k community state types. When decreasing k to 5, the case-associated 12-month community state type was combined with one of the 2-month community state types (Supplemental Figure 10). When decreasing k to 4, the control-associated community state type was also collapsed. However, case- and control-associated community state types apparent at and after the 24-month visit were still apparent. The clustering of the samples through community state types was overall fairly robust, according to the rand index, the adjusted rand index and the adjusted mutual information index (Additional file 11).

For the weighted co-occurrence network clusters, we varied the choice of normalization transform,

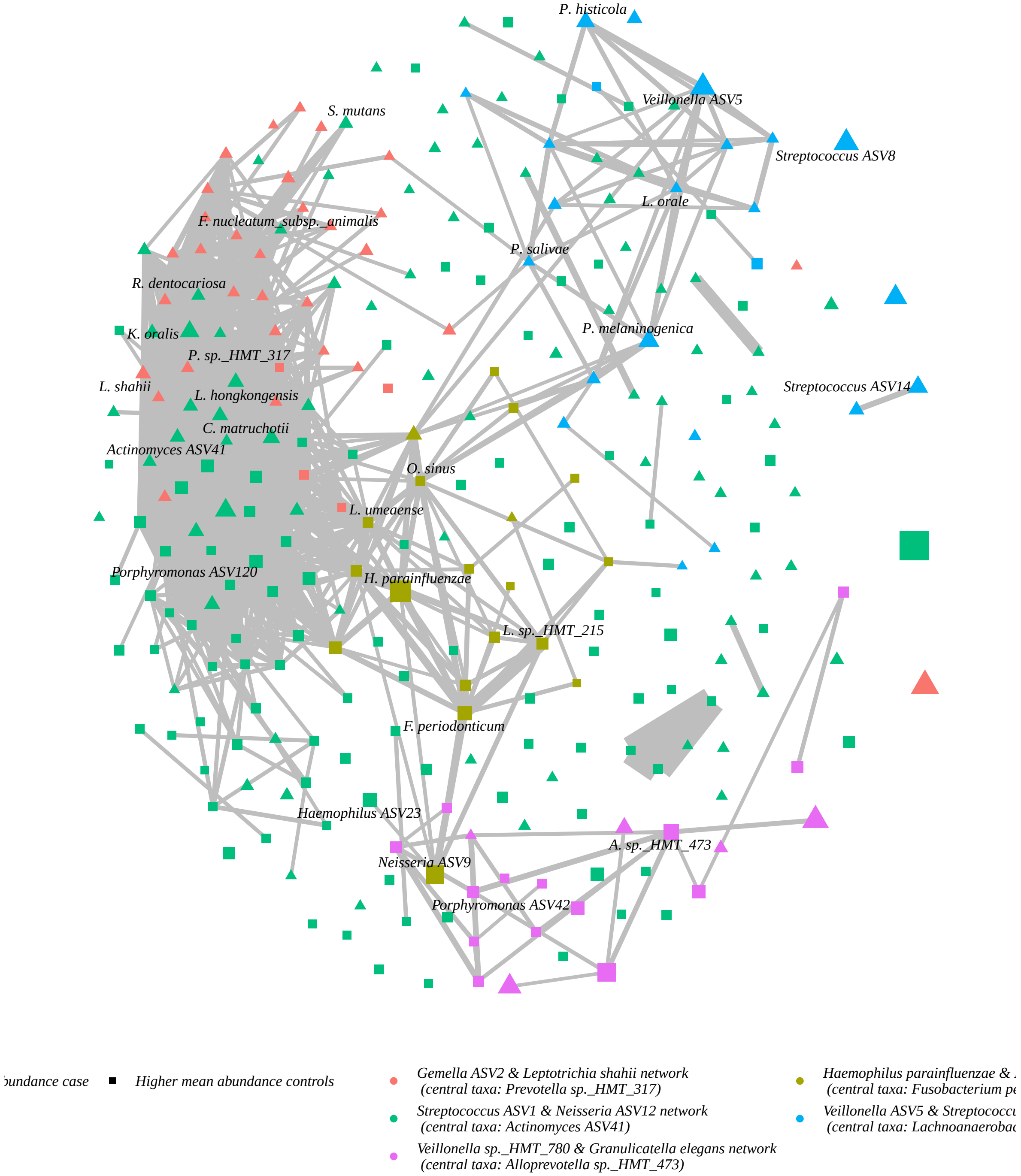

Figure 8: Structure of the entire network of ASVs, before separation into network modules

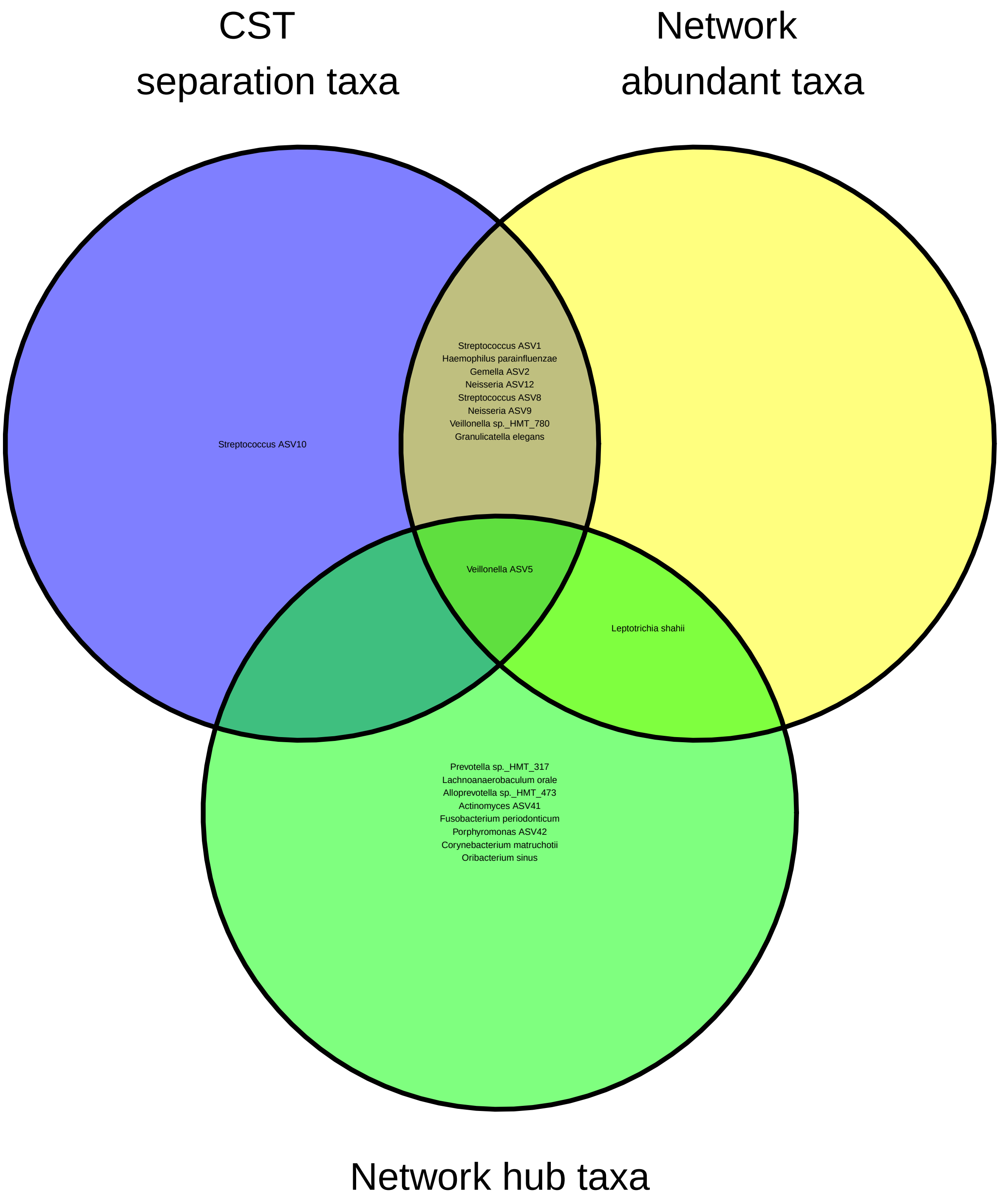

Figure 9: Venn diagram of top two ASVs in each of five network modules (10 ASVs) vs the top ten ASVs contributing to the separation of the community state types

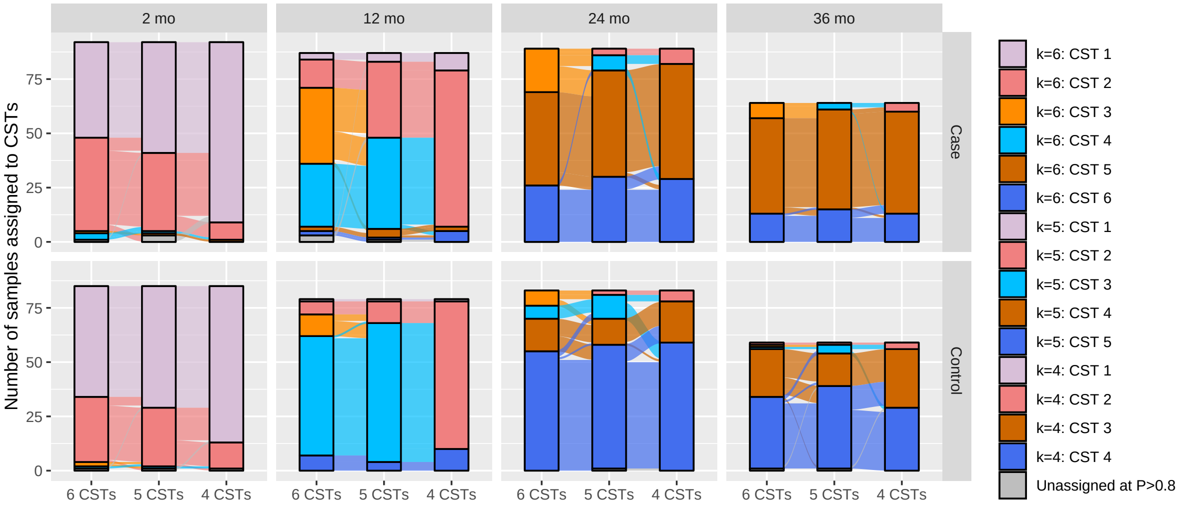

Figure 10: Sensitivity of the community state types in the COHRA2 cohort to the choice of k

comparing the Hellinger transform to the center-log ratio transform. When using the center-log ratio transform instead of the Hellinger transform, six network modules were identified instead of five. The network module with the most member taxa was split in two (Supplemental Figure 10), and some rearrangement of taxa membership existed between the other network modules, thus, clustering of the taxa across different choices of transform were better than random but not high. However, the ECC case status associated clusters (control enriched *Neisseria* ASV9-*H. parainfluenzae* network; case-enriched *Veillonella* ASV5-*Streptococcus* ASV8 network) were more stable and relatively robust (Supplemental Figure 11; Additional file 11).

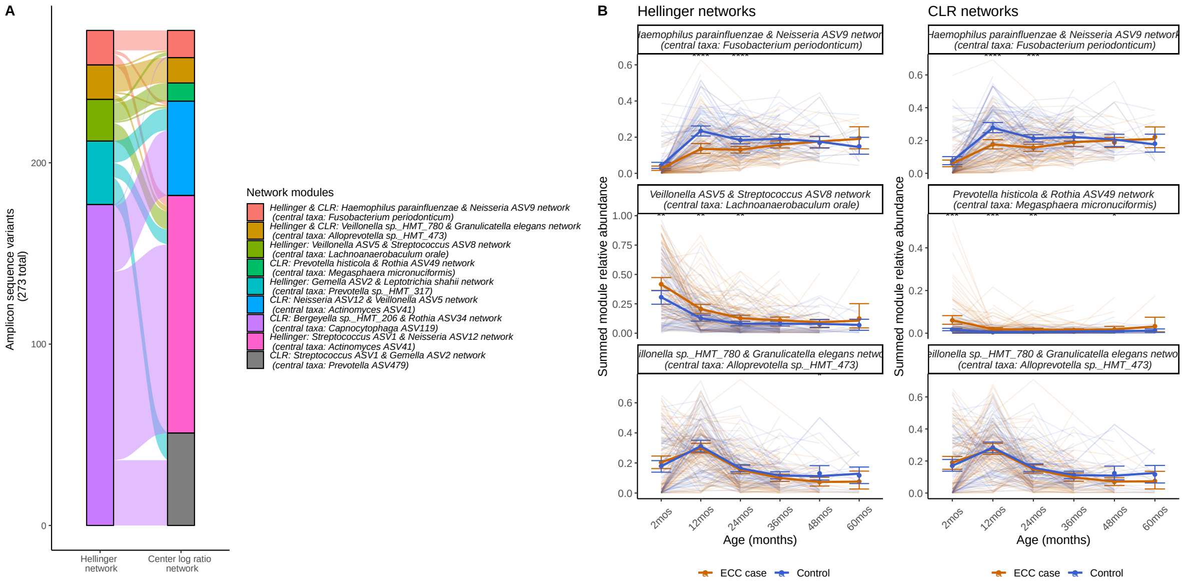

Figure 11: Sensitivity of the network modules in the COHRA2 cohort to the choice of Hellinger vs center-log ratio transform

Replication analysis

Supplemental figure 12 shows the full network modules from the Holgerson et al. cohort which were similar in composition to the ECC-associated networks in the COHRA2 cohort. Unlike in Main Figure 4, all of the edges are shown (in Main Figure 4C, edges with weights<0.03 are filtered out for visualization purposes). Notably, the protective *Haemophilus-Neisseria* network module had more nodes in the Holgerson et al. cohort than in the COHRA2 nested case-control cohort. The Holgerson et al. cohort had a much lower prevalence of ECC cases (10%) than the nested case-control cohort from COHRA2 (~50%) as a result of the design. Supplemental figure 13 shows other network modules from the Holgerson et al. cohort.

To compare the relatedness of the amplicon sequence variants assigned to various network modules across the COHRA and Holgerson cohort, we performed multiple sequence alignment of the amplicons using the R packages msa, using the ClustalW algorithim. We computed pairwise distances from the DNA sequences using the r function dist.dml from the r package phangorn, using the JC69 model. We created a neighbor joining tree using the phangorn function NJ, then fit a generalized time-reversible with gamma rate maximum likelihood tree using the neighbor joining tree as a starting point. We obtained 100 bootstrap values for the tree using bootstrap.pml and plotted the tree using ggtree and collapsed branches present in <50 of the bootstrapped trees. Supplemental Figure 14 shows bootstrapped phylogenetic trees for amplicons belonging to the A) *Neisseria* and B) *Veillonella* generas (respectively) from the Holgerson *et al.* and COHRA2 cohorts.

Multivariable sensitivity model

Dietary sugar and oral hygiene are known risk factors for ECC. The COHRA2 cohort did collect a food frequency questionnaire, however, very few children had any reported consumption of high-sugar/cariogenic foods and beverages as early as 12-months of age (Additional File 12), when we could first observe an association between the salivary microbiome and early childhood caries.

Associations between unsupervised clusters from microbiome data and count of primary teeth/age at tooh emergence

The *H. parainfluenzae & Neisseria ASV9* module did not correlate with the total number of primary teeth present at the 12- or 24-month visits, although the *Streptococcus ASV1 and Neisseria ASV12 module* did (Supplemental Figure 15). The *Veillonella ASV5* and *Streptococcus ASV8* network module was inversely correlated wtih count of primary teeth although this did not reach statistical significance. Interestingly, the *Streptococcus* ASVs that were a part of this network were closely related to *Streptococcus* species that have been previously reported to decrease after tooth emergence (see Dzidic (2018). https://doi.org/10.1038/s41396-018-0204-z). *Streptococcus* ASV8 was likely *Streptococcus salivarius*, and *Streptococcus* ASV10 was closely related to *Streptococcus lactarius/peroris* (see Additional file 4 and supplemental figure 3), both of which Dzidic et al. reported decreased from 6 months to 24 months of age. Similarly, although the mean number of primary teeth increased between community state types which were dominate at different times, the number of primary teeth did not significantly differ between the community state types that were codominant at any one time (Supplemental Figure 14). Among the subset of children recruited from Pennsylvania, data on the approximate age at first tooth emergence was available. In this subset, age at first tooth emergence was not associated with either WCN modules or CSTs.

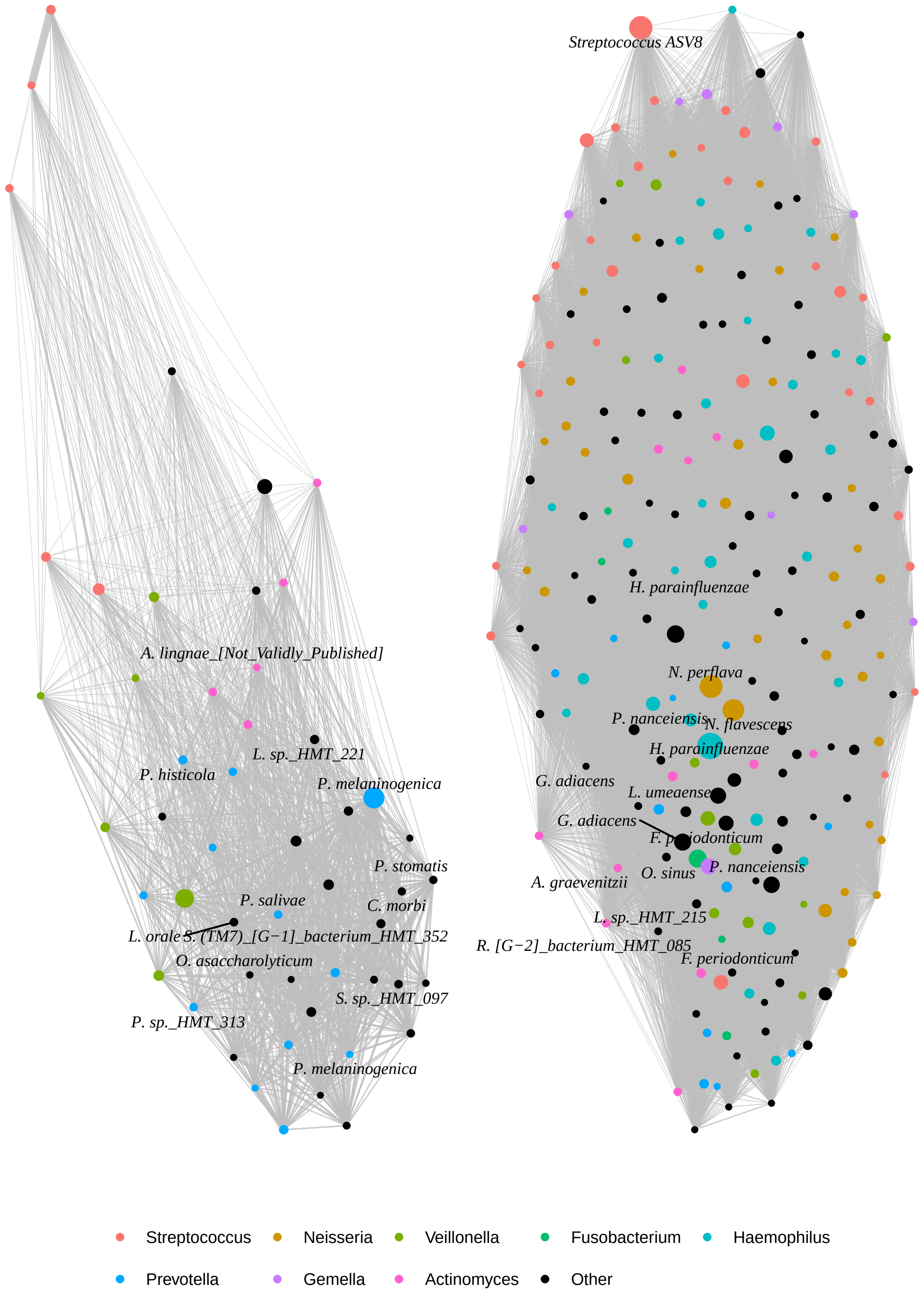

Figure 12: Network modules from the Holgerson et al. cohort that are similar in composition and strucutre to those observed in the COHRA2 cohort

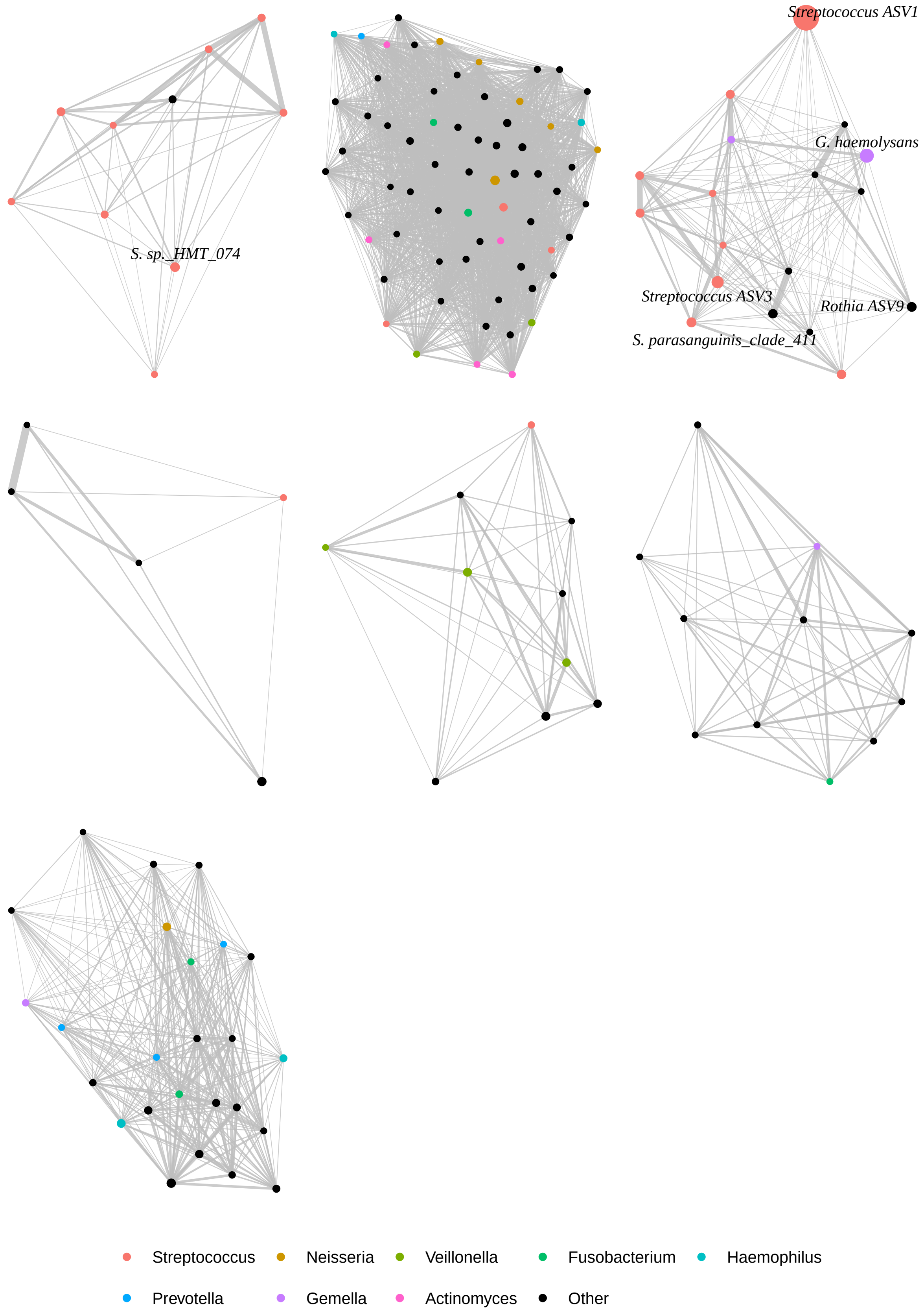

Figure 13: Other network modules identified in the Holgerson et al. cohort

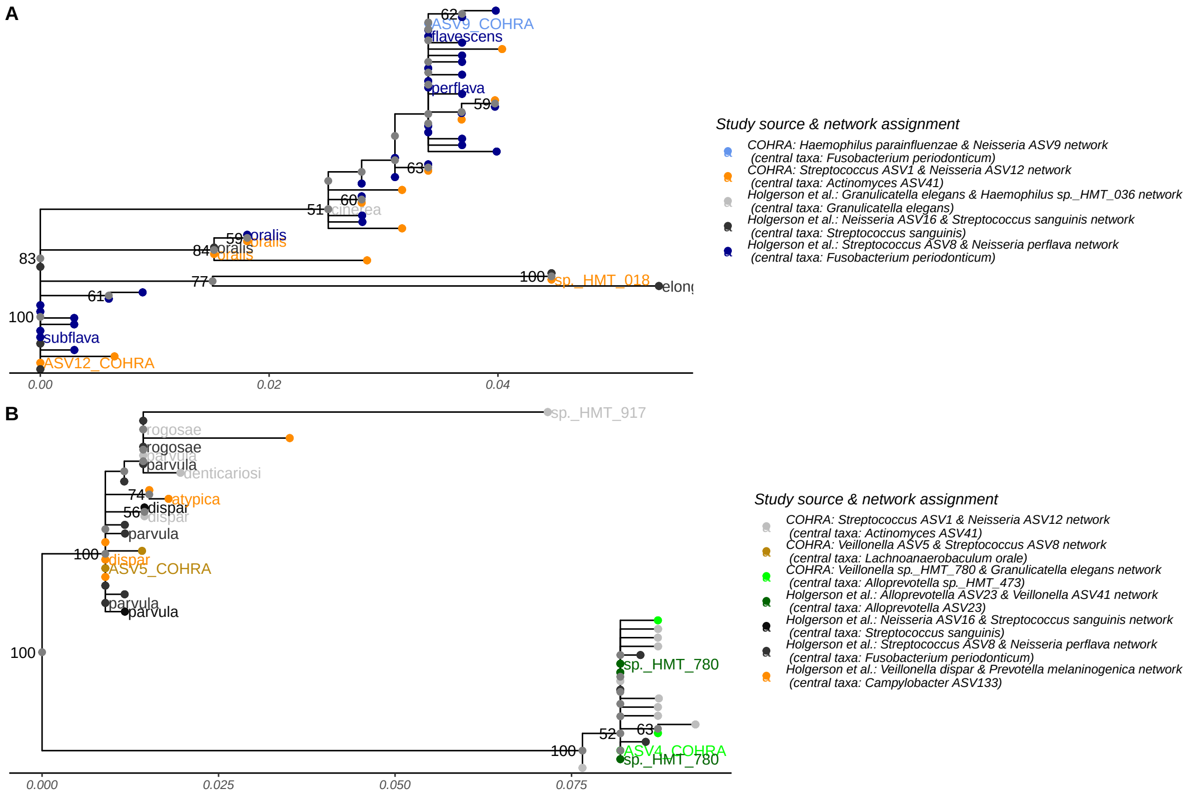

Figure 14: Phylogenetic trees showing relationships between A) Neisseria amplicons and B) Veillonella amplicons in the COHRA2 and Holgerson et al. cohorts

Whole-genome shotgun metagenomics of incident samples reveals taxonomic and functional differences between incident case- and control-samples

Volcano plot: taxa with abundances different between cases and controls in whole genome metagenome samples

Additional file 13 contains the results from the DESEQ2 analysis on the taxa from WGS sequenced plaque and saliva samples, including the log2 fold change and adjusted P values. Supplemental figure 16 presents the same information as a volcano plot. Additional file 14 contains the results from the DESEQ2 analysis of KEGG orthologs from WGS sequenced plaque and saliva samples while additional file 15 contains the result from GSEA (gene set enrichment) analysis if the KEGG pathways.

All the case enriched KEGG orthologs annotating to oxidative phosphorylation were found only in the genus Candida. For the 18 KEGG orthologs which were significantly different between cases and controls at a Benjamini-Hochberg corrected alpha of 0.05 and annotated to oxidative phosphorylation as their first KEGG pathway, additional file 16 shows the KEGG ortholog number, KEGG ortholog name, log2 fold change and all the taxonomic assignments for the contiguous sequences in which that KEGG ortholog was found. All the KEGG orthologs more abundant in cases were found only in either yeast or unclassified contiguous contigs, while the KEGG ortholgos more abundant in controls were found in bacterial species. Supplemental figure 17 shows the abundance of the species in which the

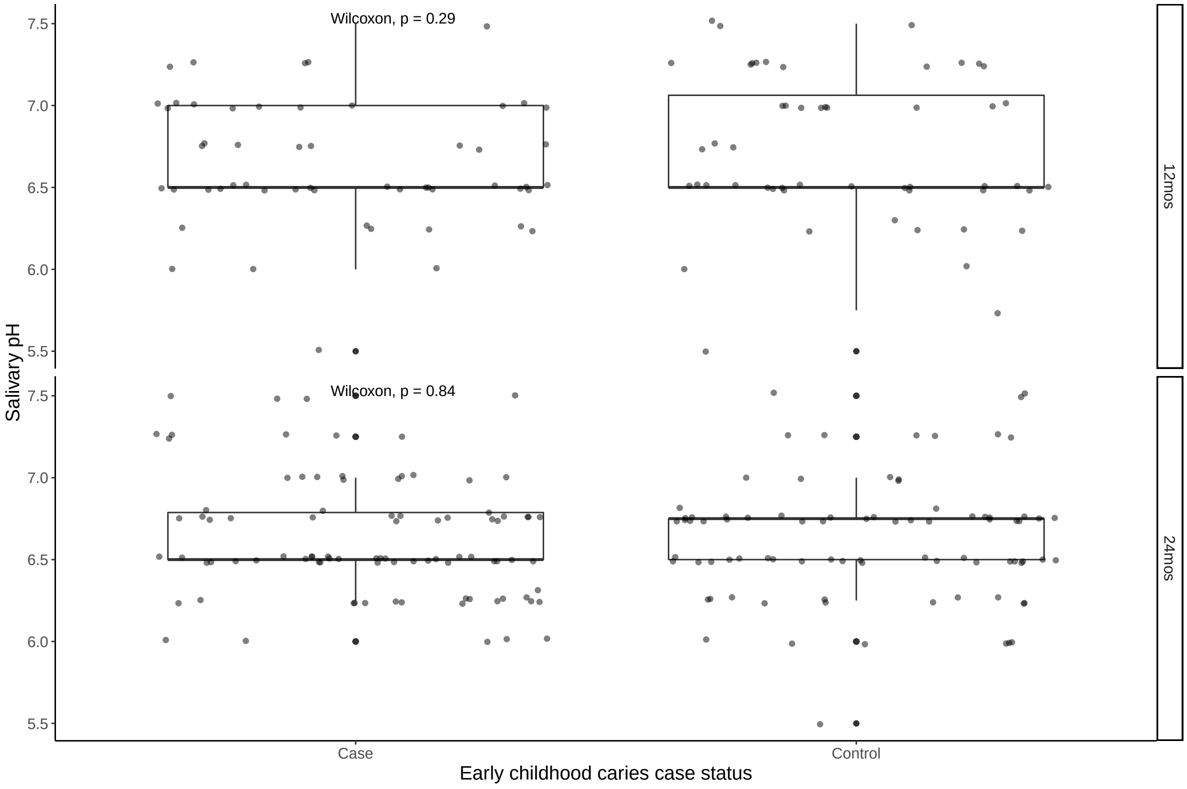

Figure 15: Salivary pH by case status among n=106 (12-months) and n=174 (24-months) pre-incident saliva samples

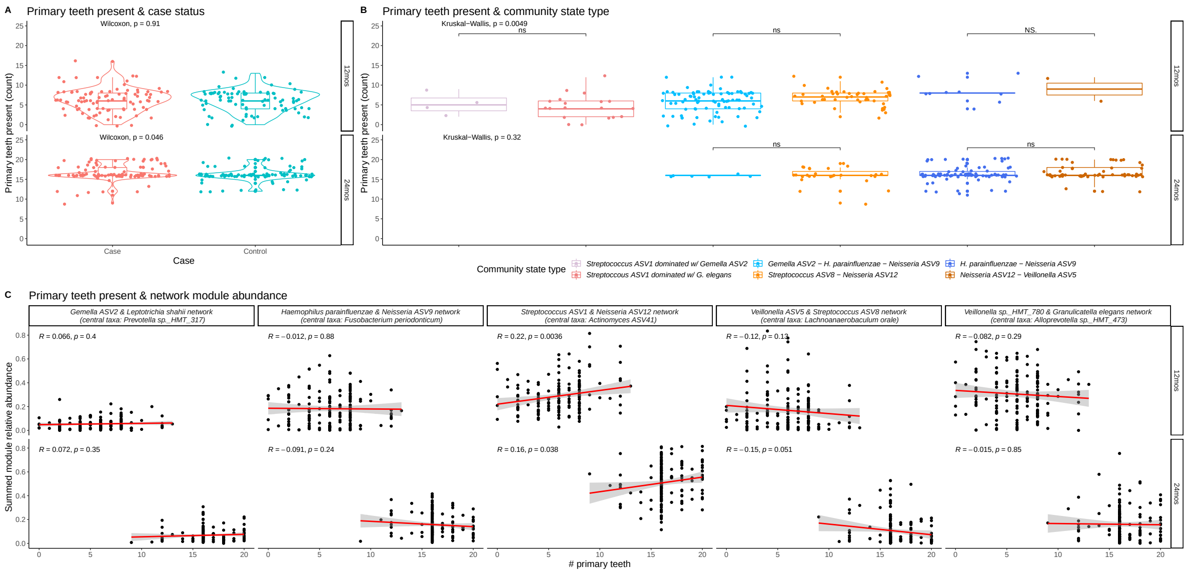

Figure 16: Relationships between unsupervised clusters and the number of primary teeth

KEGG orthologs were found.

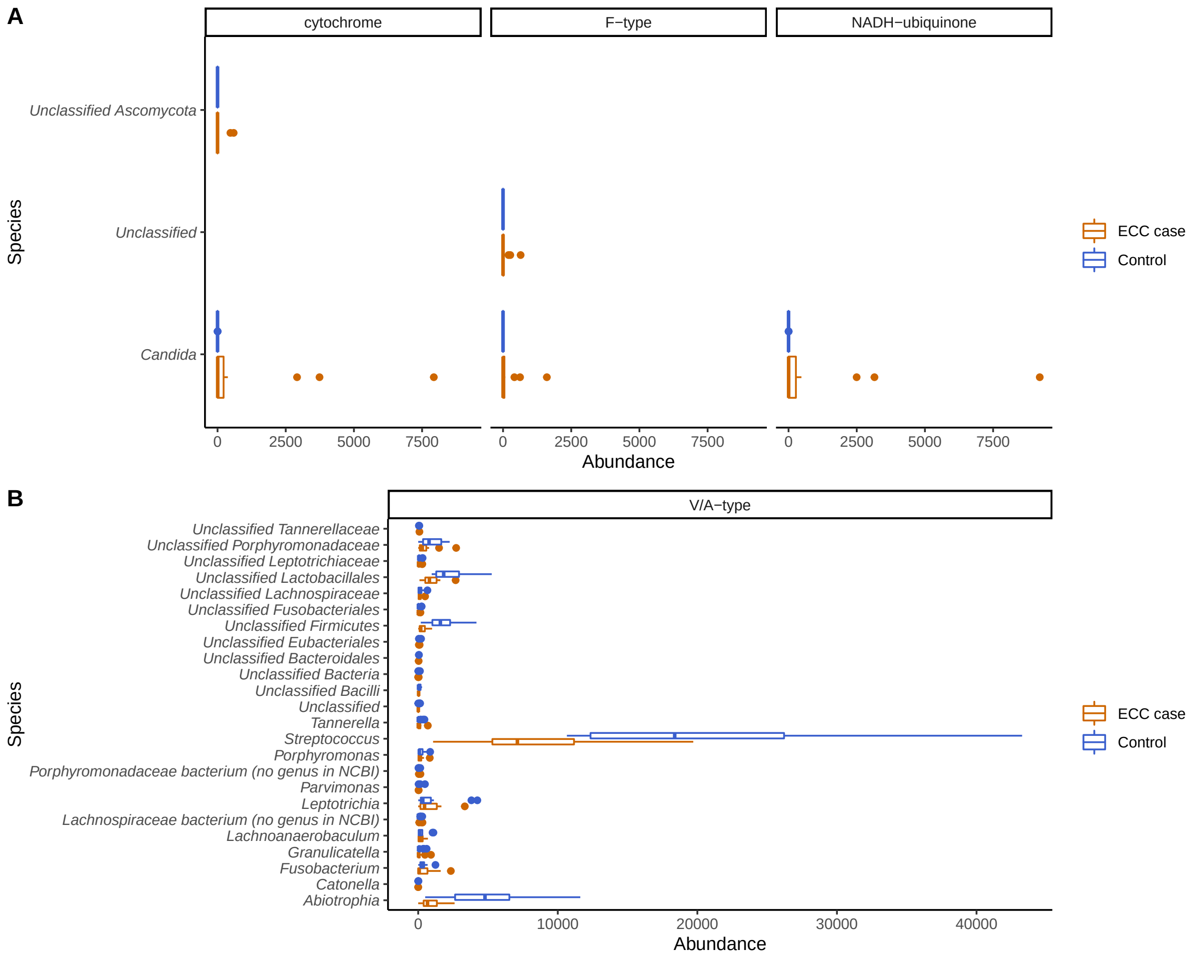

Figure 17: Abundance of taxa responsible for oxidative phosphorylation KEGG orthologs differentially abundant between cases and controls in plaque samples
