# Supplementary material for: Evaluating the ecological hypothesis: early life salivary microbiome assembly predicts dental caries in a longitudinal case-control study
Source: Microbiome. 2022 Dec 26;10:240. doi: 10.1186/s40168-022-01442-5 (PMC9791751; doi:10.1186/s40168-022-01442-5)
Supplement: Supplementary file 3 — Additional file 2: Supplementary Table. Distribution of key characteristics among non-case children in the Center for Oral Health Research in Appalachia 2 cohort and among the children sampled into the nested case-control analysis set as controls. [file 40168_2022_1442_MOESM2_ESM.docx]

|  | Distribution of key characteristics among non-case children in the Center for Oral Health Research in Appalachia 2 cohort and among the children sampled into the nested case-control analysis set as controls | | | | |
| --- | --- | --- | --- | --- | --- |
| Characteristic | N | Overall, N = 995^1^ | In nested case-control sample, N = 90^1^ | Not in nested case-control sample, N = 905^1^ | p-value^2^ |
| Child's sex | 995 |  |  |  | 0.7 |
| Female |  | 465 (47%) | 44 (49%) | 421 (47%) |  |
| Male |  | 530 (53%) | 46 (51%) | 484 (53%) |  |
| Child age (months) | 995 | 2 (0, 8) | 2 (1, 6) | 2 (0, 8) | 0.3 |
| Child's race | 990 |  |  |  | 0.010 |
| Not white |  | 216 (22%) | 10 (11%) | 206 (23%) |  |
| White |  | 774 (78%) | 80 (89%) | 694 (77%) |  |
| Unknown |  | 5 | 0 | 5 |  |
| Maternal education reported at prenatal visit | 994 |  |  |  | 0.5 |
| Associates degree or higher |  | 582 (59%) | 56 (62%) | 526 (58%) |  |
| High school degree or less |  | 412 (41%) | 34 (38%) | 378 (42%) |  |
| Unknown |  | 1 | 0 | 1 |  |
| Delivery | 990 |  |  |  | 0.8 |
| C-section |  | 277 (28%) | 26 (29%) | 251 (28%) |  |
| Vaginal |  | 713 (72%) | 64 (71%) | 649 (72%) |  |
| Unknown |  | 5 | 0 | 5 |  |
| Count of primary teeth erupted/present | 993 | 0 (0, 2) | 0 (0, 0) | 0 (0, 2) | 0.5 |
| Unknown |  | 2 | 1 | 1 |  |
| Currently breastfed | 977 |  |  |  | 0.3 |
| Currently breastfeeding |  | 571 (58%) | 57 (63%) | 514 (58%) |  |
| Not currently breastfeeding |  | 406 (42%) | 33 (37%) | 373 (42%) |  |
| Unknown |  | 18 | 0 | 18 |  |
| ^1^n (%); Median (Range) | | | | | |
| ^2^Pearson's Chi-squared test; Wilcoxon rank sum test | | | | | |
