# Supplementary material for: Evaluating the ecological hypothesis: early life salivary microbiome assembly predicts dental caries in a longitudinal case-control study
Source: Microbiome. 2022 Dec 26;10:240. doi: 10.1186/s40168-022-01442-5 (PMC9791751; doi:10.1186/s40168-022-01442-5)
Supplement: Supplementary file 4 — Additional file 3: Supplementary Table. Additional case severity statistics by 12- and 24-month microbial community state type in the COHRA2 incidence-density sampled case-control subset. [file 40168_2022_1442_MOESM3_ESM.docx]

Additional case severity statistics by 12- and 24-month microbial community state type in the COHRA2 incidence-density sampled case-control subset

|  | 12-month microbial community state type | | | | | | | 24-month microbial community state type | | | |
| --- | --- | --- | --- | --- | --- | --- | --- | --- | --- | --- | --- |
| Characteristic | *H. parainfluenzae - Neisseria ASV9, N = 9^1^* | *Streptoccous ASV1 dominated w/ G. elegans, N = 19^1^* | *Streptococcus ASV8 - Neisseria ASV12, N = 45^1^* | *Neisseria ASV12 - Veillonella ASV5, N = 2^1^* | *Streptococcus ASV1 dominated w/ Gemella ASV2, N = 4^1^* | *Gemella ASV2 - H. parainfluenzae-Neisseria ASV9, N = 84^1^* | *Unclassified, N = 3^1^* | *H. parainfluenzae - Neisseria ASV9, N = 81^1^* | *Streptococcus ASV8 - Neisseria ASV12, N = 27^1^* | *Neisseria ASV12 - Veillonella ASV5, N = 58^1^* | *Gemella ASV2 - H. parainfluenzae-Neisseria ASV9, N = 6^1^* |
| Incident visit |  |  |  |  |  |  |  |  |  |  |  |
| 12-month visit | 0 (0%) | 1 (5.3%) | 2 (4.4%) | 0 (0%) | 0 (0%) | 3 (3.6%) | 0 (0%) |  |  |  |  |
| 24-month visit | 1 (11%) | 3 (16%) | 16 (36%) | 0 (0%) | 0 (0%) | 14 (17%) | 0 (0%) | 14 (17%) | 6 (22%) | 18 (31%) | 0 (0%) |
| 36-month visit | 3 (33%) | 11 (58%) | 16 (36%) | 1 (50%) | 2 (50%) | 35 (42%) | 1 (33%) | 38 (47%) | 11 (41%) | 23 (40%) | 1 (17%) |
| 48-month visit | 5 (56%) | 3 (16%) | 9 (20%) | 1 (50%) | 2 (50%) | 19 (23%) | 1 (33%) | 20 (25%) | 8 (30%) | 13 (22%) | 4 (67%) |
| 60-month visit | 0 (0%) | 1 (5.3%) | 2 (4.4%) | 0 (0%) | 0 (0%) | 13 (15%) | 1 (33%) | 9 (11%) | 2 (7.4%) | 4 (6.9%) | 1 (17%) |
| Case/control |  |  |  |  |  |  |  |  |  |  |  |
| Case | 2 (22%) | 13 (68%) | 35 (78%) | 2 (100%) | 3 (75%) | 29 (35%) | 3 (100%) | 26 (32%) | 20 (74%) | 43 (74%) | 0 (0%) |
| Control | 7 (78%) | 6 (32%) | 10 (22%) | 0 (0%) | 1 (25%) | 55 (65%) | 0 (0%) | 55 (68%) | 7 (26%) | 15 (26%) | 6 (100%) |
| Count of primary teeth with fillings, decay, or white spots (case definition) at incident visit | 0 (0, 3) | 2 (0, 12) | 2 (0, 11) | 8 (1, 14) | 2 (0, 2) | 0 (0, 8) | 1 (1, 3) | 0 (0, 6) | 1 (0, 14) | 2 (0, 12) | 0 (0, 0) |
| Age (months) at diagnosis | 46 (29, 53) | 37 (12, 63) | 36 (11, 60) | 43 (38, 48) | 43 (37, 48) | 37 (12, 61) | 47 (36, 61) | 37 (23, 61) | 37 (23, 60) | 36 (23, 60) | 48 (38, 63) |
| Percent of present teeth with fillings, decay, or white spots (%) at incident visit | 0 (0, 15) | 15 (0, 60) | 10 (0, 55) | 38 (5, 70) | 8 (0, 10) | 0 (0, 40) | 5 (5, 15) | 0 (0, 30) | 6 (0, 70) | 11 (0, 60) | 0 (0, 0) |
| Count of primary teeth with fillings or decay (excludes white spots) at incident visit | 0 (0, 3) | 1 (0, 10) | 1 (0, 8) | 1 (0, 2) | 2 (0, 2) | 0 (0, 7) | 1 (0, 3) | 0 (0, 6) | 1 (0, 8) | 0 (0, 10) | 0 (0, 0) |
| Percent of present teeth with fillings or decay (excludes white spots, %) at incident visit | 0 (0, 15) | 5 (0, 50) | 5 (0, 40) | 5 (0, 10) | 8 (0, 10) | 0 (0, 35) | 5 (0, 15) | 0 (0, 30) | 5 (0, 40) | 0 (0, 50) | 0 (0, 0) |
| Count of primary teeth present at incident visit | 20 (16, 20) | 20 (8, 20) | 20 (6, 20) | 20 (20, 20) | 20 (20, 20) | 20 (5, 20) | 20 (20, 20) | 20 (14, 20) | 20 (12, 20) | 20 (12, 20) | 20 (20, 20) |
| ^1^n (%); Median (Range) | | | | | | | | | | | |
