# Supplementary material for: Evaluating the ecological hypothesis: early life salivary microbiome assembly predicts dental caries in a longitudinal case-control study
Source: Microbiome. 2022 Dec 26;10:240. doi: 10.1186/s40168-022-01442-5 (PMC9791751; doi:10.1186/s40168-022-01442-5)
Supplement: Supplementary file 7 — Additional file 6: Supplementary Table. Demographics of the subset with shotgun metagenomic sequenced plaque and saliva samples. [file 40168_2022_1442_MOESM6_ESM.docx]

Demographics of the subset with shotgun metagenomic sequenced plaque and saliva samples

|  | 2mos | | | 12mos | | | 24mos | | |
| --- | --- | --- | --- | --- | --- | --- | --- | --- | --- |
| Characteristic | Child has no shotgun sequenced samples from incident visit, N = 135^1^ | Child has shotgun sequenced samples from incident visit, N = 29^1^ | p-value^2^ | Child has no shotgun sequenced samples from incident visit, N = 148^1^ | Child has shotgun sequenced samples from incident visit, N = 29^1^ | p-value^2^ | Child has no shotgun sequenced samples from incident visit, N = 156^1^ | Child has shotgun sequenced samples from incident visit, N = 30^1^ | p-value^2^ |
| Site |  |  | 0.2 |  |  | 0.059 |  |  | 0.12 |
| PA | 64 (47%) | 10 (34%) |  | 69 (47%) | 8 (28%) |  | 76 (49%) | 10 (33%) |  |
| WV | 71 (53%) | 19 (66%) |  | 79 (53%) | 21 (72%) |  | 80 (51%) | 20 (67%) |  |
| Child's sex |  |  | 0.6 |  |  | 0.6 |  |  | 0.8 |
| Female | 68 (50%) | 13 (45%) |  | 74 (50%) | 13 (45%) |  | 76 (49%) | 14 (47%) |  |
| Male | 67 (50%) | 16 (55%) |  | 74 (50%) | 16 (55%) |  | 80 (51%) | 16 (53%) |  |
| Child's race |  |  | 0.2 |  |  | 0.3 |  |  | 0.3 |
| Bi- or Multi-racial | 19 (14%) | 1 (3.4%) |  | 16 (11%) | 1 (3.4%) |  | 18 (12%) | 1 (3.3%) |  |
| White | 116 (86%) | 28 (97%) |  | 132 (89%) | 28 (97%) |  | 138 (88%) | 29 (97%) |  |
| Delivery |  |  | 0.5 |  |  | 0.4 |  |  | 0.5 |
| C-section | 46 (34%) | 12 (41%) |  | 50 (34%) | 12 (41%) |  | 51 (33%) | 12 (40%) |  |
| Vaginal | 88 (66%) | 17 (59%) |  | 97 (66%) | 17 (59%) |  | 104 (67%) | 18 (60%) |  |
| Unknown | 1 | 0 |  | 1 | 0 |  | 1 | 0 |  |
| Maternal education reported at prenatal visit |  |  | 0.8 |  |  | 0.5 |  |  | 0.7 |
| Associates degree or higher | 61 (45%) | 14 (48%) |  | 66 (45%) | 15 (52%) |  | 71 (46%) | 15 (50%) |  |
| High school degree or less | 74 (55%) | 15 (52%) |  | 82 (55%) | 14 (48%) |  | 85 (54%) | 15 (50%) |  |
| SalivapH | 6 (6, 8) | 6 (6, 7) | 0.3 | 6 (6, 8) | 7 (6, 8) | 0.6 | 6 (6, 8) | 6 (6, 8) | 0.8 |
| Unknown | 84 | 20 |  | 60 | 11 |  | 7 | 3 |  |
| Count of primary teeth erupted/present | 0 (0, 2) | 0 (0, 0) | 0.5 | 6 (0, 16) | 6 (0, 12) | >0.9 | 16 (9, 20) | 16 (14, 20) | >0.9 |
| Case |  |  | 0.6 |  |  | 0.6 |  |  | 0.5 |
| Case | 68 (50%) | 13 (45%) |  | 79 (53%) | 14 (48%) |  | 83 (53%) | 14 (47%) |  |
| Control | 67 (50%) | 16 (55%) |  | 69 (47%) | 15 (52%) |  | 73 (47%) | 16 (53%) |  |
| Maternal report of child antibiotics within 3 mos prior to visit | 13 (9.6%) | 3 (10%) | >0.9 | 39 (26%) | 7 (24%) | 0.8 | 47 (30%) | 5 (17%) | 0.13 |
| Visit of case diagnosis |  |  | 0.001 |  |  | 0.002 |  |  | 0.002 |
| 12mos | 5 (3.7%) | 0 (0%) |  | 7 (4.7%) | 0 (0%) |  | 7 (4.5%) | 0 (0%) |  |
| 24mos | 40 (30%) | 0 (0%) |  | 40 (27%) | 0 (0%) |  | 41 (26%) | 0 (0%) |  |
| 36mos | 50 (37%) | 16 (55%) |  | 55 (37%) | 18 (62%) |  | 59 (38%) | 17 (57%) |  |
| 48mos | 27 (20%) | 9 (31%) |  | 33 (22%) | 7 (24%) |  | 36 (23%) | 9 (30%) |  |
| 60mos | 13 (9.6%) | 4 (14%) |  | 13 (8.8%) | 4 (14%) |  | 13 (8.3%) | 4 (13%) |  |
| Currently breastfed |  |  | 0.4 |  |  | >0.9 |  |  | >0.9 |
| Currently breastfeeding | 73 (54%) | 13 (45%) |  | 41 (28%) | 8 (28%) |  | 10 (6.4%) | 1 (3.3%) |  |
| Not currently breastfeeding | 62 (46%) | 16 (55%) |  | 107 (72%) | 21 (72%) |  | 146 (94%) | 29 (97%) |  |
| ^1^n (%); Median (Range) | | | | | | | | | |
| ^2^Pearson's Chi-squared test; Fisher's exact test; Wilcoxon rank sum test | | | | | | | | | |
