# Supplementary material for: Evaluating the ecological hypothesis: early life salivary microbiome assembly predicts dental caries in a longitudinal case-control study
Source: Microbiome. 2022 Dec 26;10:240. doi: 10.1186/s40168-022-01442-5 (PMC9791751; doi:10.1186/s40168-022-01442-5)
Supplement: Supplementary file 13 — Additional file 12: Food frequency of high-sugar foods and tooth brushing/wiping data by case status and child visit. [file 40168_2022_1442_MOESM12_ESM.docx]

Food frequency of high-sugar foods and tooth brushing/wiping data by case status and child visit.

|  | 2-month visit | | | 12-month visit | | | 24-month visit | | |
| --- | --- | --- | --- | --- | --- | --- | --- | --- | --- |
| Characteristic | Case, N = 99^1^ | Control, N = 91^1^ | p-value^2^ | Case, N = 93^1^ | Control, N = 84^1^ | p-value^3^ | Case, N = 99^1^ | Control, N = 92^1^ | p-value^3^ |
| [In the past 7 days, about how often did your child drink...] 100% Juice (apple, orange...)? [*NO ADDED WATER*] |  |  | 0.8 |  |  | 0.002 |  |  | 0.015 |
| Never or once | 64 (94%) | 47 (96%) |  | 33 (37%) | 54 (65%) |  | 17 (18%) | 30 (34%) |  |
| Every few days | 0 (0%) | 0 (0%) |  | 22 (24%) | 11 (13%) |  | 17 (18%) | 24 (27%) |  |
| Once a day | 1 (1.5%) | 1 (2.0%) |  | 16 (18%) | 10 (12%) |  | 26 (28%) | 15 (17%) |  |
| Several times a day | 3 (4.4%) | 1 (2.0%) |  | 19 (21%) | 8 (9.6%) |  | 32 (35%) | 20 (22%) |  |
| Unknown | 31 | 42 |  | 3 | 1 |  | 7 | 3 |  |
| [In the past 7 days, about how often did your child drink...] Soda/pop (Carbonated soft drinks) |  |  | >0.9 |  |  | 0.4 |  |  | 0.051 |
| Never or once | 68 (100%) | 49 (100%) |  | 85 (94%) | 82 (99%) |  | 76 (82%) | 85 (94%) |  |
| Every few days | 0 (0%) | 0 (0%) |  | 3 (3.3%) | 1 (1.2%) |  | 8 (8.6%) | 3 (3.3%) |  |
| Once a day | 0 (0%) | 0 (0%) |  | 2 (2.2%) | 0 (0%) |  | 7 (7.5%) | 2 (2.2%) |  |
| Several times a day | 0 (0%) | 0 (0%) |  | 0 (0%) | 0 (0%) |  | 2 (2.2%) | 0 (0%) |  |
| Unknown | 31 | 42 |  | 3 | 1 |  | 6 | 2 |  |
| [In the past 7 days, about how often did your baby eat...] Desserts, pastries, cookies, cakes, ice cream, popsicles? |  |  | >0.9 |  |  | 0.6 |  |  | 0.4 |
| Never or once | 5 (100%) | 3 (100%) |  | 58 (64%) | 60 (72%) |  | 26 (28%) | 26 (29%) |  |
| Every few days | 0 (0%) | 0 (0%) |  | 20 (22%) | 14 (17%) |  | 41 (44%) | 44 (49%) |  |
| Once a day | 0 (0%) | 0 (0%) |  | 11 (12%) | 9 (11%) |  | 25 (27%) | 16 (18%) |  |
| Several times a day | 0 (0%) | 0 (0%) |  | 1 (1.1%) | 0 (0%) |  | 1 (1.1%) | 3 (3.4%) |  |
| Unknown | 94 | 88 |  | 3 | 1 |  | 6 | 3 |  |
| [In the past 7 days, about how often did your baby eat...] Candies, fruit snacks? |  |  | >0.9 |  |  | 0.6 |  |  | 0.2 |
| Never or once | 5 (100%) | 3 (100%) |  | 87 (97%) | 82 (99%) |  | 59 (63%) | 61 (69%) |  |
| Every few days | 0 (0%) | 0 (0%) |  | 3 (3.3%) | 1 (1.2%) |  | 23 (25%) | 16 (18%) |  |
| Once a day | 0 (0%) | 0 (0%) |  | 0 (0%) | 0 (0%) |  | 10 (11%) | 7 (7.9%) |  |
| Several times a day | 0 (0%) | 0 (0%) |  | 0 (0%) | 0 (0%) |  | 1 (1.1%) | 5 (5.6%) |  |
| Unknown | 94 | 88 |  | 3 | 1 |  | 6 | 3 |  |
|  |  |  |  |  |  | 0.3 |  |  | >0.9 |
| Does not brush/wipe teeth | 1 (100%) | 1 (100%) |  | 9 (10%) | 12 (15%) |  | 2 (2.2%) | 3 (3.3%) |  |
| Unknown | 98 | 90 |  | 5 | 2 |  | 6 | 2 |  |
| Brushes/Wipes teeth, with toothpaste |  |  |  | 37 (42%) | 26 (32%) |  | 87 (94%) | 83 (92%) |  |
| Brushes/Wipes teeth, without toothpaste |  |  |  | 42 (48%) | 44 (54%) |  | 4 (4.3%) | 4 (4.4%) |  |
| ^1^n (%) | | | | | | | | | |
| ^2^Fisher's exact test | | | | | | | | | |
| ^3^Pearson's Chi-squared test; Fisher's exact test | | | | | | | | | |
